# Mechanism and Protective Effect of *Smilax glabra* Roxb on the Treatment of Heart Failure *via* Network Pharmacology Analysis and Vitro Verification

**DOI:** 10.3389/fphar.2022.868680

**Published:** 2022-05-23

**Authors:** Yingxin Long, Zunjiang Li, Chunxia Huang, Zhongyu Lu, Kuncheng Qiu, Meixing He, Zhijian Fang, Banghan Ding, Xiaohong Yuan, Wei Zhu

**Affiliations:** ^1^ The Second Clinical College, Guangzhou University of Chinese Medicine, Guangzhou, China; ^2^ Department of Emergency, Panyu Hospital of Traditional Chinese Medicine, Guangzhou, China; ^3^ Guangdong Provincial Hospital of Traditional Chinese Medicine, Guangzhou, China

**Keywords:** *Smilax glabra* Roxb, heart failure, mitochondrial dysfunction, network pharmacology analysis, UHPLC-LTQ-Orbitrap-MSn method

## Abstract

*Smilax glabra* Roxb (SGR) has been widely applied alone or in combination with other Chinese herbs in heart failure (HF), but its mechanism and protective effect have not been investigated. We aimed to explore the mechanism and protective effect of SGR on the treatment of HF. Network pharmacology analysis predicted that SGR was involved in the regulation of cell proliferation, oxidation–reduction process, apoptotic process, ERK1 and ERK2 cascade, MAPK cascade, etc. Its mechanism was mainly involved in the MAPK signaling pathway, calcium signaling pathway, cardiac muscle contraction, etc. Subsequently, SGR was proved to improve cellular viability, restore cellular morphology, suppress cellular and mitochondrial ROS production, improve H_2_O_2_-induced lysosome inhibition, attenuate mitochondrial dysfunction, and protect mitochondrial respiratory and energy metabolism in H9c2 cells. SGR activated the p38MAPK pathway by decreasing the mRNA expression of AKT, PP2A, NF-KB, PP2A, RAC1, and CDC42 and increasing the mRNA expression of Jun, IKK, and Sirt1. SGR also decreased the protein expression of ERK1, ERK2, JNK, Bax, and Caspase3 and increased the protein expression of p38MAPK and Bcl-2. In addition, Istidina at the highest degree was identified in SGR *via* the UHPLCLTQ-Orbitrap-MSn method, and it was suggested as anti-heart failure agents by targeting SRC with molecular docking analysis. In conclusion, SGR has a protective effect on HF through cellular and mitochondrial protection *via* multi-compounds and multi-targets, and its mechanism is involved in activating the p38 MAPK pathway. Istidina may be possible anti-HF agents by targeting SRC.

## GRAPHICAL ABSTRACT

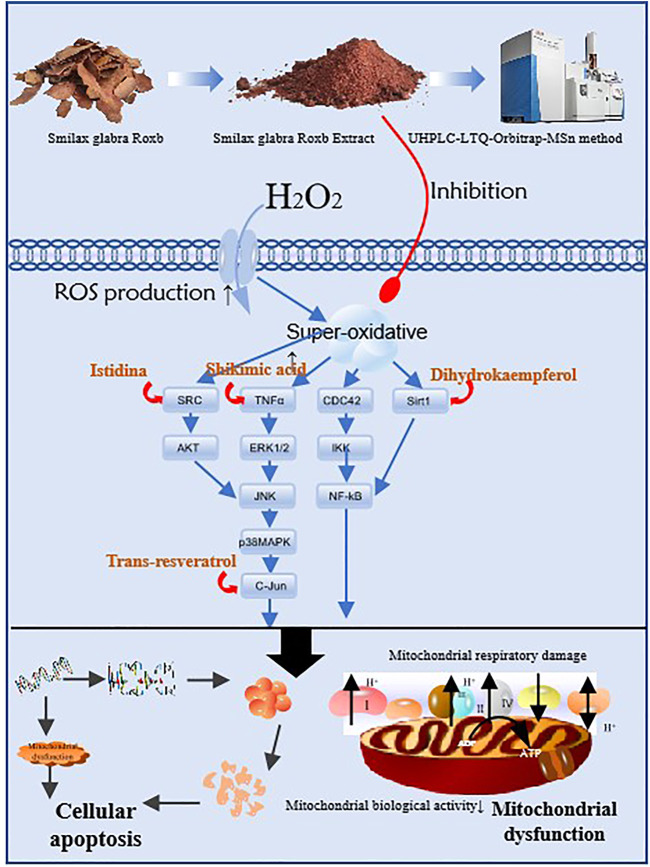

## Highlights

(1) For the first time, the mechanism of *Smilax glabra* Roxb (SGR) on heart failure was predicted by network pharmacology analysis
(2) For the first time, SGR was proved to exert a protective effect on cellular injury and mitochondrial dysfunction with vitro verification
(3) For the first time, SGR was proved as a multi-target agent with multi-compounds for heart failure *via* the UHPLC-LTQ-Orbitrap-MSn method and molecule docking analysis
(4) Five compounds with potential protective in total glycosides from SGR were identified, and Istidina was first considered as possible anti-heart failure agent

## Introduction

Heart failure (HF), a multifactorial and systemic disease, is responsible for more than 17.3 million deaths each year, and its mortality ranks top in cardiovascular diseases (Chen et al., 2018). Mitochondrial dysfunction and cellular damage induced by oxidative stress are important pathophysiological mechanisms for the occurrence and development of HF (Kiyuna et al., 2018; Zhou and Tian, 2018). Excessive accumulation of reactive oxygen species (ROS) induced by oxidative stress in the heart damages the targeted DNA, proteins, lipids, and other components of cardiomyocytes, leading to lowering energy production in mitochondria and reducing cardiac output of the heart (Li et al., 2006; Van Der Pol et al., 2019). Thus, the protection of mitochondrial function and the regulation of reactive oxygen species are critical for HF treatment (Zhou and Tian, 2018).

It is known that mitogen-activated protein kinases (MAPKs), one of the most important downstream signaling pathways of ROS, are involved in cellular responses to external stimuli *via* regulating the activity of cellular proteins, enzymes, and transcription factors (Aggeli et al., 2006). The mammalian MAPK family contains three major members: p38 MAPKs (p38), c-Jun N-terminal kinase (JNK), and extracellular signal-regulated kinases (ERKs). A previous study proved that activation of Jun, p38, and ERKs was helpful for the myocardium (Duan et al., 2020). ERK1/2 is involved in cellular differentiation and proliferation, the JNK cascade regulates cell differentiation, apoptosis, and inflammation, and the p38 cascade may be involved in apoptosis and regulating cytokine responses (Wang et al., 2018; Guo et al., 2020). Oxidative stress leads to JNK phosphatase activation and triggers antioxidation by various enzymes; for example, SOD dismutase promotes O^2−^ catalysis to H_2_O_2_, but glutathione peroxidases can degrade H_2_O_2_ (Li et al., 2006). Oxidative stress activates the extracellular ERK signaling pathway and its downstream molecule to clear excessive ROS, which is the key process involved in regulating myocardial apoptosis when oxidative stress occurs in cardiomyocytes (Gong et al., 2019).

*Smilax glabra* Roxb (SGR) has been applied in the treatment of cardiovascular diseases clinically for more than 2,000 years in China (Hao et al., 2017). The anti-viral, anti-inflammatory, immunomodulatory activities and anti-oxidative damage potentials of SGR were extensively studied, but its effect on the cardiovascular system has been rarely reported (Kwon et al., 2020). Recent research proved that SGR and its extracted compound Aiphanol exerted a pharmacological effect on the cardiovascular system *via* blocking angiogenesis and tumor growth factor. Aiphanol also directly inhibited VEGFR2 and COX2 (Chen et al., 2021). SGR has been reported to have a potential effect on myocardial hypertrophy treatment (Cai et al., 2015). Lei-gong-gen formula granule can promote vasodilation and reduce blood pressure in a spontaneously hypertensive rat model (Li et al., 2021). SGR is served as the most important TCM in this formula (Wang et al., 2015). However, there are few reports about SGR in the treatment of HF.

In the present study, network pharmacology analysis was conducted to investigate the active components of SGR and predict its underlying mechanism on HF. Then, the protective effect of SGR was verified by vitro experiments in the H_2_O_2_-induced cardiomyocytes model. Finally, the UHPLC-LTQ-Orbitrap-MSn technique and molecule docking analysis were applied to identify the possible targets of screened active compounds responsible for HF.

## Materials and Methods

### Collection of Compounds for *Smilax glabra* Roxb

The Traditional Chinese Medicine Systems Pharmacology Database (TCMSP, http://lsp.nwu.edu.cn/), the Encyclopedia of Traditional Chinese Medicine Database (ETCM, http://www.tcmip.cn/ETCM/index.php/Home/Index/), and the TCMID Database (http://119.3.41.228:8000/tcmid) were used to collect the chemical compounds of SGR, the candidate compounds with oral bioavailability (OB) ≥ 30% and drug-likeness (DL) ≥ 0.18 were identified as active compounds through the Symmap Database (https://www.symmap.org/) indicating the compounds had chemical stability and solubility.

### Prediction of Compound-Related Targets

SGR active compounds were screened after duplication, and they were imported into the Batman Database (http://bionet.ncpsb.org/batman-tcm/index.php/Home/Index/index) to search and predict the targets of each active compound.

### Collection of Human Heart Failure Disease Targets and Venn Analysis

The original HF disease targets were obtained from the Genecards Database (https://www.genecards.org/) with a relevance score no less than 5. Then the intersection targets between HF diseases targets and predicted targets of SGR compounds were screened by Venn analysis. The intersection targets were identified as targets of SGR compounds that had a potential pharmacological effect on HF.

### Networks Construction

The active compounds of SGR and their predicted targets were introduced into Cytoscape 3.1.1 software to construct a network diagram, revealing the distribution characteristics of SGR active compounds and their targets. The network topology analysis was performed to screen the key active compounds of SGR according to the connection degree.

### Protein–Protein Interaction Data

Protein–Protein Interaction (PPI) network of the intersection targets was obtained from Search Tool for the Retrieval of Interacting Genes/Proteins (STRING, a free biological database) (http://string-db.org/; version 11.0), with the species limited to “*Homo sapiens*,” PPI network was then imported into Cytoscape 3.1.1 software for network analysis of the core subsystem. Key targets (Hub-Target) were screened according to the connection degree after network topology analysis.

### Gene Ontology Annotation and Kyoto Encyclopedia of Genes and Genomes Pathway Enrichment Analysis

Gene Ontology Annotation enrichment analysis (GO) of screened hub targets was performed *via* DAVID Database (https://david.ncifcrf.gov), including cellular component (CC), molecular function (MF), and biological process (BP). Kyoto Encyclopedia of Genes and Genomes (KEGG) pathway enrichment analysis was conducted *via* the “org.Hs.eg.db” package in R software (Version 3.6.3). Enrichment bubbles were constructed through the OMICShare tool online platform (http://www.omicshare.com/tools).

### UHPLC-LTQ-Orbitrap-MSn Analysis

Reference standards of Istidina, resveratrol, shikimic acid, epicatechin, and dihydrokaempferol were accurately weighed and dissolved with 80% methanol to obtain standard solutions at a concentration of 0.153, 0.110, 0.068, 0.065, and 0.098 mg/ml, respectively. Also, the standard solutions were filtered with a 0.22 syringe filter before analysis. A UHPLC system coupled with LTQ Orbitrap XL high resolution mass spectrometric conditions (Thermo Fisher, Scientific, United States) was applied for LC-MS analysis. The chromatographic separation was performed on an ACQUITY UPLC HSS T3 Column (2.1 × 100 mm, 1.8 µm) at a flow rate of 0.2 ml/min. The mobile phases were composed of solution A (water) and solution B (methanol) and the gradient elution was set as follows: 10% B (0–8 min), 10–35% B (8–15 min), 35–90% B (15–40 min), and 90–10% D (46–50 min). The injection volume was 4 µl. MS analysis was performed on a mass spectrometer equipped with an ESI source and the parameters were as follows: spray voltage, 3.8 KV; capillary temperature, 350.05°C; capillary voltage, −18.00 units; tube lens voltage, −67.68 V; sweep gas, 0.24 units; sheath gas and auxiliary gas were set as 29.95 and 4 units, respectively. Orbitrap mass analyzer was set up with the scan range of m/z 90–1,500 and the resolution of 30,000. The samples used negative ion mode analysis.

### Verification by Molecular Docking on the Interaction Between Key Compounds of SGR and the Hub Targets of Heart Failure

The protein structures of screened targets were downloaded from the PDB Database (https://www.rcsb.org/) and were preprocessed by PyMol software. The structural files of the key active compounds of SGR were downloaded from the PubChem Database (https://pubchem.ncbi.nlm.nih.gov/) and were saved in *mol2 format. The abovementioned structures were added hydrogens, and the charges were calculated by autodocktools1.5.6 software, respectively; then, they were saved in a PDBQT format. Finally, molecules and proteins were docked through the Autodock Vina 1.1.2 software, the model with the smallest binding energy value was selected, and their structural visualizations were conducted by PyMOL tools.

### Reagents and Antibodies

*Smilax glabra* Roxb was purchased from Kangmei Industry (LOP: 160505781). H9c2 rat cardiomyocytes (ATCC) were purchased from American Type Culture Collection (Rockville, MD, United States). Dulbecco’s Modified Eagle’s Medium, fetal bovine serum, 100  U/ml penicillin, and 100 μg/ml streptomycin were purchased from Gibco (Grand Island, NY, United States). Reactive oxygen species assay kit, mitochondrial permeability transition pore assay kit, Lyso-Tracker Red, and antifade mounting medium with DAPI were purchased from Beyotime (Beyotime Biotechnology Co. Ltd., Shanghai, China). Cell Meter™ Mitochondrial Hydroxyl Radical Detection Kit was purchased from AAT Bioquest. TMRE Mitochondrial Membrane Potential Assay Kit was purchased from Solarbio (Solarbio, Beijing, China). 3-(4,5-dimethylthiazol-2-yl) -2,5-diphenyltetrazolium bromide (MTT) was purchased from Sigma-Aldrich (MTT, Sigma-Aldrich, United States), Seahorse XF Assay Kit was purchased from Agilent (Agilent, United States). Primers were ordered from Invitrogen (Thermo Fisher). Antibodies including β-actin (ab182651), Bcl-2 (ab32124), HRP-conjugated goat anti-rabbit (ab6721), and anti-mouse IgG (ab6789) were purchased from Abcam Co. P38 MAPK (CST, 9212), SAPK/JNK (CST, 9252), and p44/42 MAPK (CST, 9102) were purchased from Cell Signaling Technology. Goat Anti-Rabbit IgG AF 488 (abmart, M21012F) was purchased from Abmart (Abmart, Shanghai).

### Cell Culture and Treatment

H9c2 cells were cultured in Dulbecco’s Modified Eagle’s Medium with 10% fetal bovine serum, 100  U/ml penicillin, and 100 μg/ml streptomycin in a 37°C, 5% CO_2_ incubator. Cells in the H_2_O_2_ group were exposed to 600 μM H_2_O_2_ for 3 h. In the SGR group, cells were pretreated with 200, 400, and 800 μg/ml SGR for 24 h, then they were exposed to 600 μM H_2_O_2_ for another 3 h. Cells in the control group were cultivated with a normal medium.

### MTT Assay

Cell viability was detected by MTT assay. In brief, H9c2 cardiomyocytes (1  ×  10^4^/well) were seeded into 96-well plates. After treatment, 10 μl of 5 mg/ml MTT solution was added to each well and then cells were incubated in the dark for 4 h. After removing the cultured medium, the crystals were dissolved in 150 μl of DMSO. The absorbance was measured at 490 nm with a BioTek plate reader (BioTek).

### Measurement of Lactate Dehydrogenase Production and Cellular Morphology Observation

Lactate dehydrogenase (LDH) production was measured by using LDH cytotoxicity Assay Kit for detection of cytotoxicity in H9c2 cells, according to the manufacturer’s protocol. Absorbance was measured at 490 nm using a BioTek plate reader (BioTek), and the values were directly proportional to the enzyme activity. In cellular morphology observation, cellular morphology was imaged by using a fully automatic inverted fluorescence microscopic analysis system (ECLIPSE Ti2-E, Nikon, Japan).

### Measurement of Superoxide Dismutase Inhibition Rate

Superoxide Dismutase (SOD) activity was measured by using Superoxide Dismutase Assay Kit for the measurement of SOD inhibition rate in H9c2 cells, according to the manufacturer’s protocol. Absorbance was measured at 450 nm using a BioTek plate reader (BioTek), and the values were directly proportional to the SOD Inhibition rate.

### Detection of Cellular ROS Production

The level of cellular ROS production was determined by using Reactive oxygen species Assay Kit, according to the manufacturer’s protocol. After treatment, the H9c2 cells were stained with DCFH-DA (1 μM) and incubated in the dark for 30 min, and then were washed three times with PBS. The cellular ROS fluorescence intensity was determined using Flow Cytometry Assay (Agilent ACEA, Quanteon, United States).

### Confocal Imaging of the Cell Meter™ Mitochondrial Hydroxyl Radical

Cell Meter™ Mitochondrial Hydroxyl Radical was detected by using MitoROS™ OH580. After treatment, cells were stained with MitoROS™ OH580 probe for 1 h at 37°C and washed twice with pre-warmed PBS, and then 100 µl Assay Buffer was added into each group and 5 μl DAPI was added for 5 min in the incubator subsequently. The intensity of fluorescence was imaged by using a laser scanning confocal microscope (Zeiss LSM710, Germany). Cell Meter™ Mitochondrial Hydroxyl Radical was represented as red fluorescence.

### Confocal Imaging of the Mitochondrial Membrane Potential (ΔΨm)

Mitochondrial membrane potential (ΔΨm) was detected using TMRE. In brief, after the indicated treatments, cells were stained with a TMRE probe (0.2 µM), for 30 min at 37°C and washed once with pre-warmed DMEM without serum. The fluorescence intensity of cells was detected by using a laser scanning confocal microscope (Zeiss LSM710, Germany). The degree of TMRE fluorescence was considered as an indication of the change of mitochondrial membrane potential.

### Detection of Opening of the Mitochondrial Permeability Transition Pore

A mitochondrial permeability transition pore assay kit was used to detect the opening of mPTP. After treatment, the culture medium was aspirated and cells were washed twice with PBS, 1 ml Calcein AM staining solution [containing 1 µl Calcein AM (×1,000), 10 µl Solvent promoting (×100), and 10 µl CoCl_2_ (×100)] were added to each sample, and cells were incubated for 30 min in the dark. After incubation, Calcein AM staining solution was aspirated, cells were washed twice with PBS, and then 500 µl detection buffer was added to the sample and the opening of mPTP was analyzed by using a BD FACSCalibur cytometer (Becton Dickinson, San Jose, CA, United States).

### Measurement of H_2_O_2_-Induced Lysosome Mediated in H9c2 Living Cells

The level of lysosome was determined using Lyso-Tracker Red according to the manufacturer’s protocol. After treatment, the H9c2 cells were stained with Lyso-Tracker Red (1 μM) solution and incubated in the dark for 10 min, then the Lyso-Tracker Red (1 μM) solution was replaced with fresh DMEM. The lysosome fluorescence intensity was determined using Flow Cytometry Assay (Agilent ACEA, Quanteon, United States).

### Evaluation of Mitochondrial Respiration

Cellular oxygen consumption rate (OCR) indicating the mitochondrial respiration was measured by using the Seahorse XF24 Extracellular Flux analyzer and software (Yu et al., 2019). Firstly, H9c2 cells were cultured in XF24 cell culture plates (1 × 10^4^ cells/well). Before the assay, 1 mmol/L pyruvate, 2 mmol/L glutamine, and 10 mmol/L glucose were added into Seahorse XF DMEM for preparation. After treatment, the cell medium was replaced with 500 μl prepared Seahorse XF DMEM, and then cells were incubated at 37°C without CO_2_ for 1 h. Thereafter, cells were exposed to oligomycin (15 Mm, complex V inhibitor), FCCP (10 μM, respiratory uncoupler), and rotenone plus antimycin A (5 μM, inhibitors of complex I and complex III) by using the XF Cell Mito Stress Test kit (Seahorse Bioscience) to measure the parameters of OCR. Finally, the OCR value was normalized by CCK8 analysis. Respiration rates were calculated as the mean of all determinations at a certain state.

### Immunofluorescence Staining

H9c2 cardiomyocytes were seeded in a laser confocal dish at a density of 1 × 10^5^ cells/ml (200 μl per dish). After treatment, the cell culture medium was removed, cells were washed three times with DPBS and fixed with 4% paraformaldehyde for 15 min. Then, paraformaldehyde was removed, cells were washed three times with PBS and blocked with BSA for 15 min, subsequently, cells were incubated with primary antibody Bcl-2 overnight at 4°C. Thereafter, cells were incubated with fluorescent secondary antibody (1:3,000) for 1 h after washing three times with PBS. 5 μl DAPI was added for 5 min in the incubator before imaging by using a fully automatic inverted fluorescence microscopic analysis system (ECLIPSE Ti2-E, Nikon, Japan).

### Real-Time Quantitative Polymerase Chain Reaction Analysis

Real-time quantitative polymerase chain reaction (qRT-PCR) was used to evaluate the mRNA expression level of pathway genes. Total RNA was isolated from H9c2 cells using Trizol reagent, according to the standard protocol, and RNA concentration was determined by using a NanoDrop spectrophotometer (Thermo Scientific, Rockford, IL, United States). 3 μg of total RNA was employed for reverse-transcription to yield cDNA. Subsequently, the synthesized cDNA was amplified using a 10 μl reaction system by the SYBR master Mixture (TAKARA, Japan). Finally, the threshold cycle (CT) value and the relative mRNA expression of the genes were calculated. The sequences of primers were shown as follows Table 1:

**TABLE 1:** Primer sequences for qRT-PCR.

Gene	Forward (5′-3′)	Reserve (5′-3′)
β-active	CTG​AGA​GGG​AAA​TCG​TGC​GTG​AC	AGG​AAG​AGG​ATG​CGG​CAG​TGG
Sirt1	CTT​CTT​GGA​GAC​TGC​GAT​G	TTG​TGT​TCG​TGG​AGG​TTT​T
Akt1	GCT​GGA​GAA​CCT​CAT​GCT​G	GTGTCCCGCAGAACGTC
RAC1	CATCCTAGTGGGGACGAA	AGG​TAT​TTG​ACA​GCA​CCG​A
JUN	GGAGCCAACCAACGTGA	GTC​CCC​GCT​TCA​GTA​ACA​A
IKK	GCAAATGAGGACCAGAGC	CGCAGGAAAGATGACCAC
NF-KB	GGGCAGAAGTCAACGCT	TGTCGTCCCATCGTAGGT
CDC42	GTGTGCTGCTATGAACGC	TATTACCGGAAGGGCAAG
ARRB	GCCTGCGGTGTGGATTA	CCA​GAG​ATG​CCT​CAA​GGT​G
PPAR	ACCCTTTACCACGGTTGA	CAG​GCT​CTA​CTT​TGA​TCG​C
PP2A1	TGTATCAGTGGGCACGAC	AGG​AGT​TGC​CAG​AGA​GGT​T
PKC	ATCCCTGCTGCGTTCCT	TTG​TGG​CTC​TTC​ACC​TCG​T
JNK	TAG​AGC​ATC​CCA​GTC​TTC​G	CAAGCCAGGTGTGAACG

### Western Blot Analysis

After the intervention, H9c2 cells were harvested and the total protein was extracted by using RIPA Lysis Buffer (Beyotime Biotechnology Co., Ltd., Shanghai, China) with pierce protease inhibitor tablet (Thermo Scientific, Rockford, IL, United States) and pierce phosphatase inhibitor tablet (Thermo Scientific, Rockford, IL, United States). Then total protein was quantified and mixed with 5X SDS-PAGE protein buffer (Beyotime Biotechnology Co., Ltd., Shanghai, China). After electrophoresis, the separated proteins were transferred to a polyvinylidene difluoride (PVDF) membrane. Later, the membranes were incubated with 5% non-fat milk in TBST (Tris-buffered saline with 0.1% Tween-20) for 2 h at room temperature, washed three times with TBST, and then the membrane with target proteins was incubated overnight at 4°C with primary antibody (1:1,000) against Bax, Caspase-3, p38 MAPK, JNK, ERK, β-actin, and Tubulin overnight at 4°C. Thereafter, the membrane was incubated with a respective HRP conjugated secondary antibody at 1:3,000 for 1 h after washing three times with TBST. BioRad imaging system (BioRad, Hercules, CA, United States) was applied to detect the fluorescent signal and the signal strip was quantified using Image Lab Software (BioRad, Hercules, CA, United States).

### Statistical Analysis

The experimental data, obtained from at least three independent experiments, were presented as the mean ± SEM. Statistical analysis was performed by one-way ANOVA analysis followed by the Bonferroni test for multiple comparisons by using GraphPad Prism software, version 8.0 for Windows (GraphPad Software Inc., United States). A probability of less than 0.05 was defined as statistically significant.

## Results and Discussion

### Collection of Chemical Ingredients and Targets in *Smilax glabra* Roxb

**FIGURE 1:**
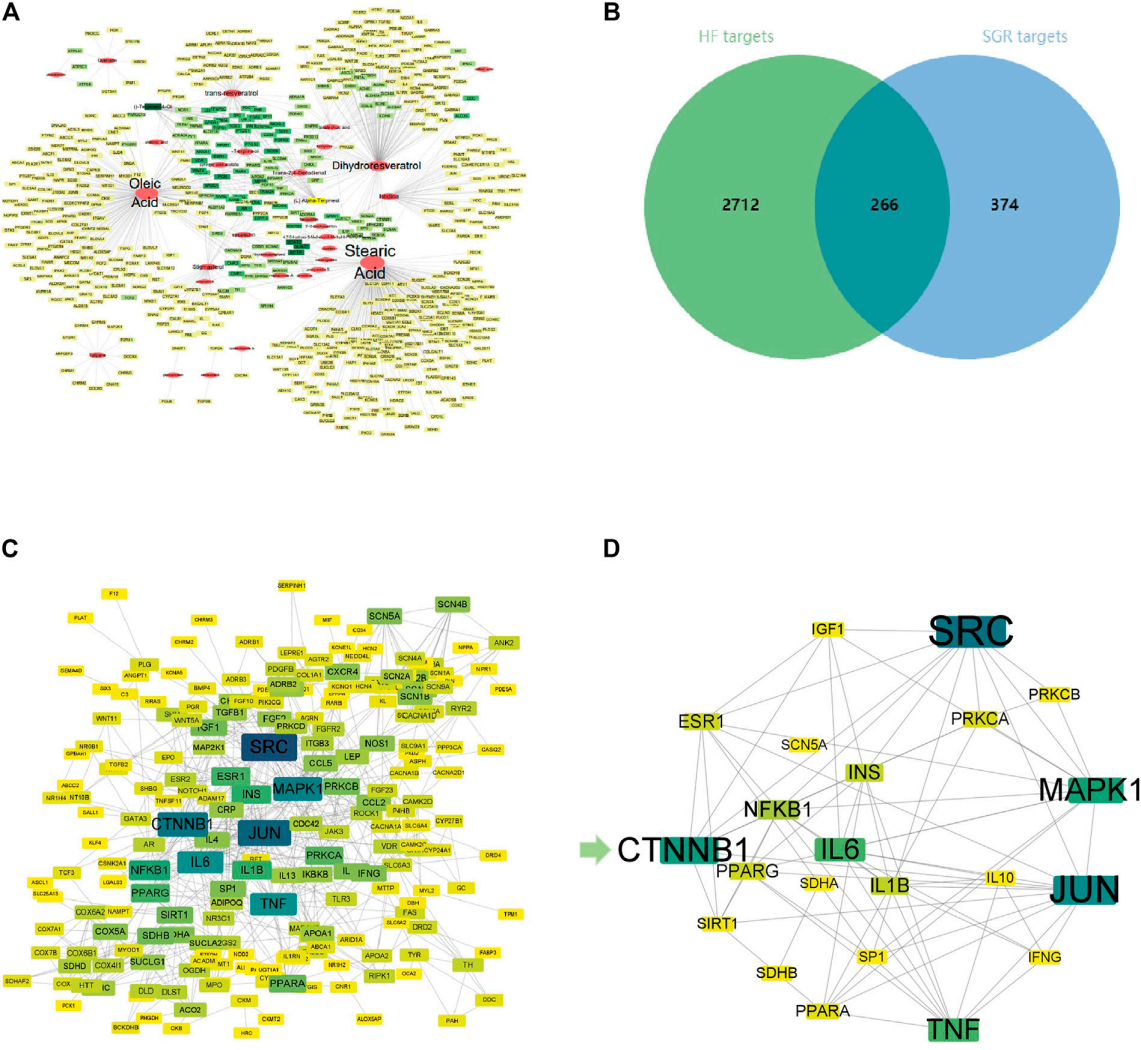
Activated Chemical Composition of SGR and predicted Target Corresponding Feature Network Diagram. **(A)** Distribution characteristic between 32 active components of SGR and their predicted targets; **(B)** Venn analysis of targets between DEGs of heart failure and SGR candidate targets; **(C)** protein–protein interaction (PPI) network of intersection targets; and **(D)** 25 hub targets with node degrees two-fold greater than the average node degree. The nodes get larger with an increasing degree. Edges: PPIs between targets of cryptotanshinone (CPT) and their interaction partners; shallower color circle nodes: common targets of GSR and heart failure (HF); Deep-color circle nodes: hub targets of GSR and HF. GO Enrichment Analysis *via* DAVID Database.

According to the TCMSP, ETCM, and TCMID Database, 77 chemical ingredients in SGR were collected, then 55 active ingredients of SGR were screened through the Symmap Database with the condition of OB ≥ 30%.Then, 32 active compounds were selected after removing ethylene ingredients, volatile oil ingredients, and duplicate ingredients (Details in Supplementary Table S1). Finally,a total of 640 predicted targets of 32 active components of SGR were collected (Details in Supplementary Table S2). Active components–predicted targets network was constructed to screen the hub active components of SGR (Details in Supplementary Table S3). As shown in Figure 1A, this network consisted of 689 nodes and 902 edges. The average degree node of the common components was 28.19 . In total, 5 hub components were identified, whose node degrees were far greater than the average node degree in this network. The nodes interacted with others *via* numerous edges (226 in Stearic Acid, 183 in Oleic Acid, 128 in Dihydroresveratrol, 62 in trans-resveratrol, and 42 in Istidina). The results of this network suggested that these five hub components may be the potential compounds of SGR in the treatment of HF.

### Identification of Targets Acting on HF From SGR Candidate Targets

A total of 2,978 HF diseases targets with relevance scores no less than 5 were selected after duplication (Details in Supplementary Table S4), then 266 intersection targets were screened *via* Venn analysis between SGR candidate targets and HF targets (as Figure 1B). The intersection targets were considered as the targets acting on HF from SGR candidate targets (Details in Supplementary Table S5).

### Protein–Protein Interaction Network Analysis

A PPI network of 266 intersection targets was constructed to illustrate the interactions between targets, as shown in Figure 1C, this network consisted of 232 nodes and 680 edges. The average degree node of the common targets was 5.86. In total, 25 hub targets were identified (Figure 1D), whose node degrees were two-fold greater than the average node degree in this network (38 in SRC, 34 in JUN, 31 in CTNNB1, 29 in MAPK1, 26 in IL6, 26 in TNF, 20 in NFKB1, 18 in IL1B, 18 in INS, 17 in ESR1, 17 in PPARG, 16 in PRKCA, 15 in PPARA, 14 in IGF1, 14 in SIRT1, 14 in PRKCB, 14 in SDHB, 13 in IL10, 13 in IFNG, 13 in SP1, and 13 in SCN5A) (Detailed in Supplementary Table S6).

**FIGURE 2:**
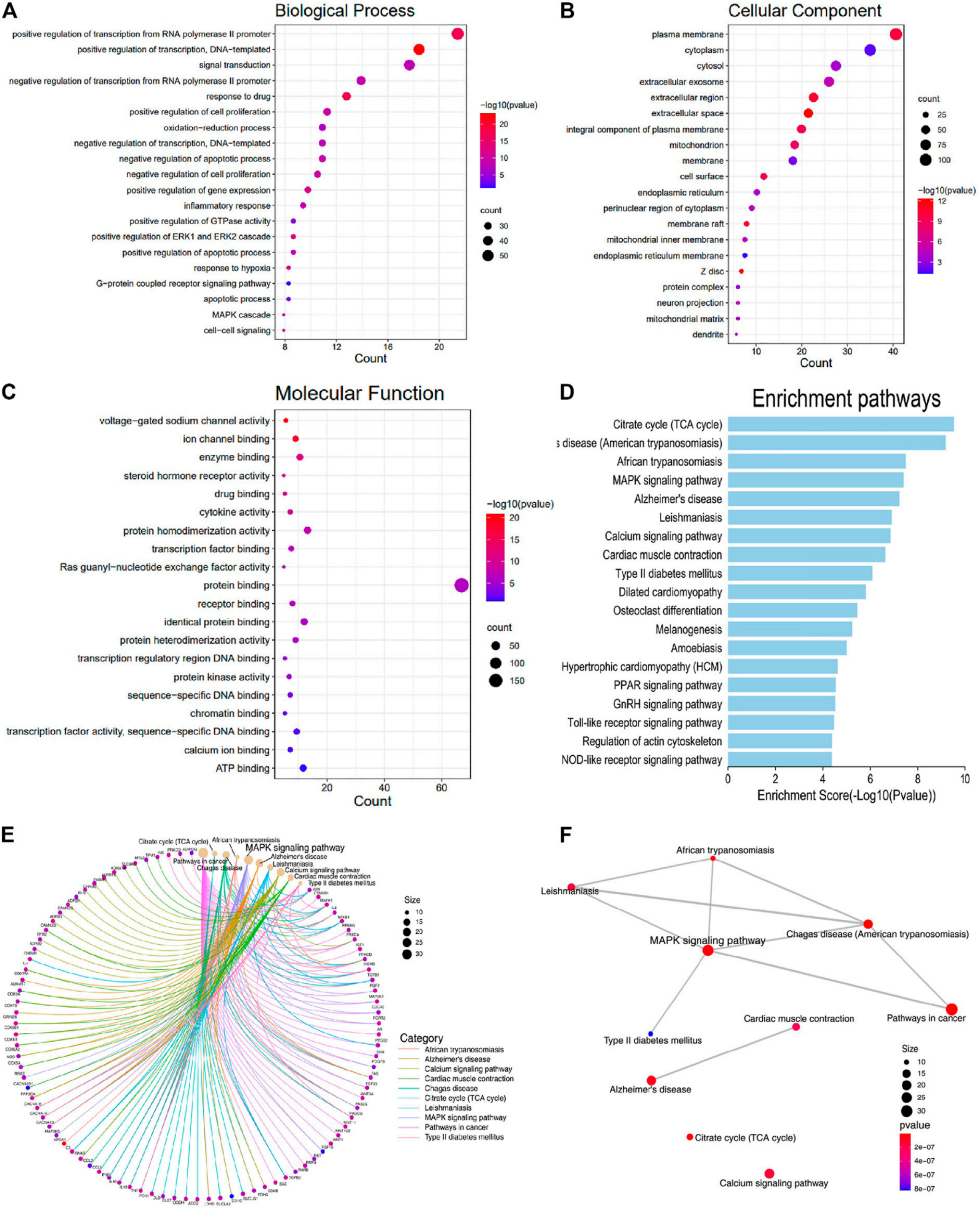
Associated targets were enriched in the functional module of Biological processes (BP), Cellular component (CC), Molecular function (MF), and KEGG pathway enrichment. **(A)** Biological processes (BP); **(B)** cellular component (CC); **(C)** molecular function (MF); **(D)** KEGG enrichment of the intersection targets between SGR predicted targets and heart failure; **(E)** gene-concept network analysis on KEGG enrichment; and **(F)** enrichment map analysis on KEGG enrichment.

GO enrichment analysis was conducted to investigate the enrichment of the associated targets in biological processes (BP), cellular component (CC), and molecular function (MF) using the DAVID Database (detailed in Supplementary Table S7). The associated targets enriched in CC included cytoplasm, cytosol, mitochondrion, cell surface, endoplasmic reticulum, mitochondrial inner membrane, and endoplasmic reticulum membrane (Figure 2B). MF enrichment mainly included protein binding, ATP binding, enzyme binding, and receptor binding (Figure 2C). As shown in Figure 2A, the main functional modules in BP included regulation of transcription, cell proliferation, oxidation-reduction process, apoptotic process, ERK1 and ERK2 cascade, response to hypoxia, MAPK cascade, and inflammatory response. Previous studies have validated that MAPK participates in cell proliferation and apoptosis, mitochondrial regulation, and heart function, but there is no literature exploring the relationship between SGR and MAPK signaling pathway, and the pharmacodynamics of SGR.

### KEGG Enrichment Analysis on Intersection Targets

**FIGURE 3:**
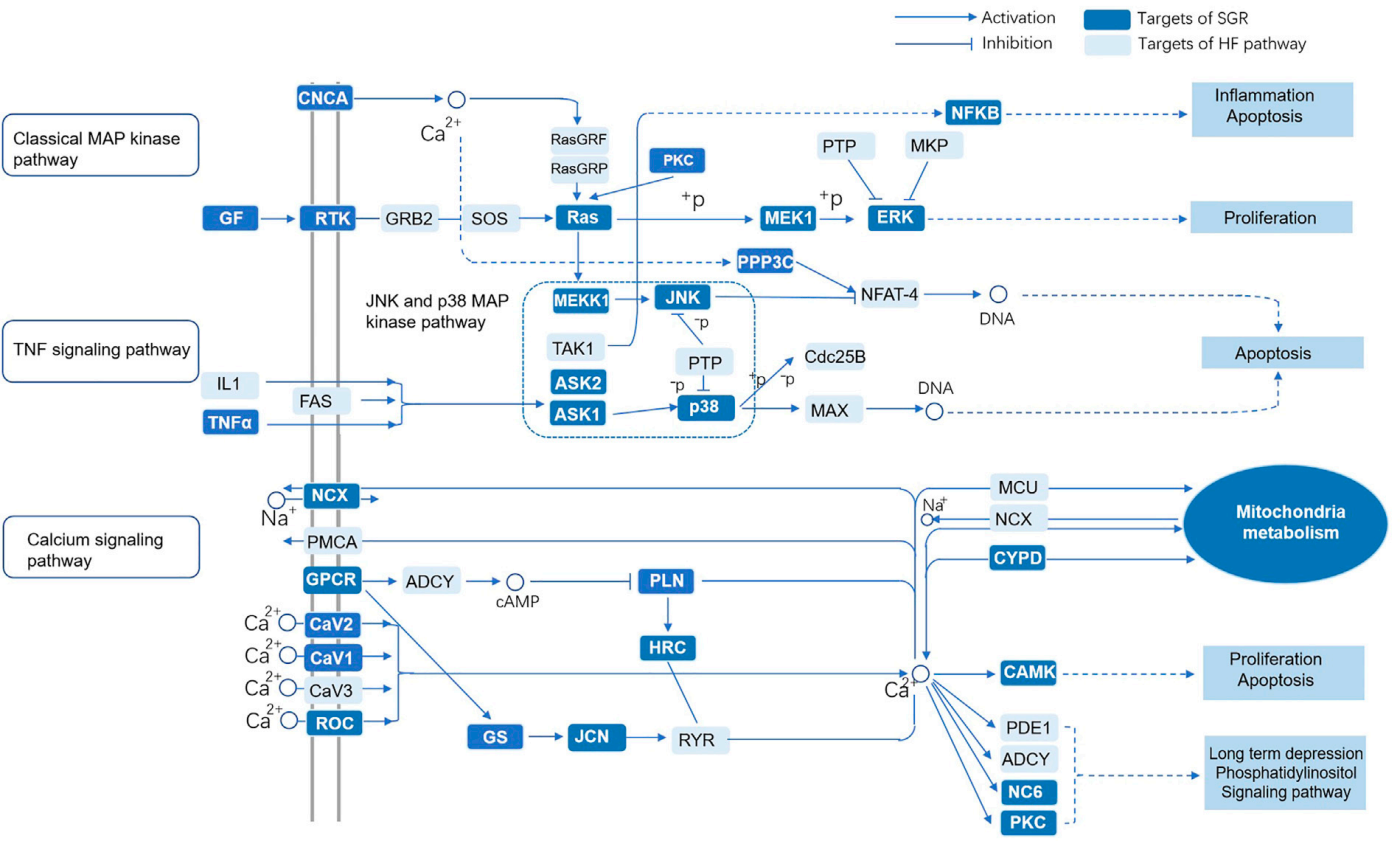
“HF-pathway” network of the predicted targets of SGR enriched in pathways correlated with HF. *Smilax glabra* Roxb protected cardiomyocytes against H_2_O_2_-induced cytotoxicity.

KEGG enrichment analysis was used to explore the molecular mechanism of SGR involving heart failure treatment (Detailed in Supplementary Table S8). The mechanisms mainly included MAPK signaling pathway, Alzheimer’s disease, calcium signaling pathway, cardiac muscle contraction, type II diabetes mellitus, dilated cardiomyopathy, hypertrophic cardiomyopathy (HCM), PPAR signaling pathway, GnRH signaling pathway, toll-like receptor signaling pathway, and NOD-like receptor signaling pathway. (Figure 2D). When we conducted a gene-concept network (Figure 2E) and enrichment map analysis (Figure 2F), it all indicated that the MAPK signaling pathway was the potential mechanism of the SGR. The intersection targets of SGR enriched in pathways correlated with HF were integrated into an “HF-pathway” network, as Figure 3 showed, the MAPK signaling pathway and aforementioned pathways were involved in the regulation of a variety of biological processes including cell proliferation, apoptosis, inflammation, and mitochondrial metabolism, all of which were closely associated with HF progression. Thus, we further verified whether the MAPK signaling pathway is involved in the pharmacologic effect of GSR.

**FIGURE 4:**
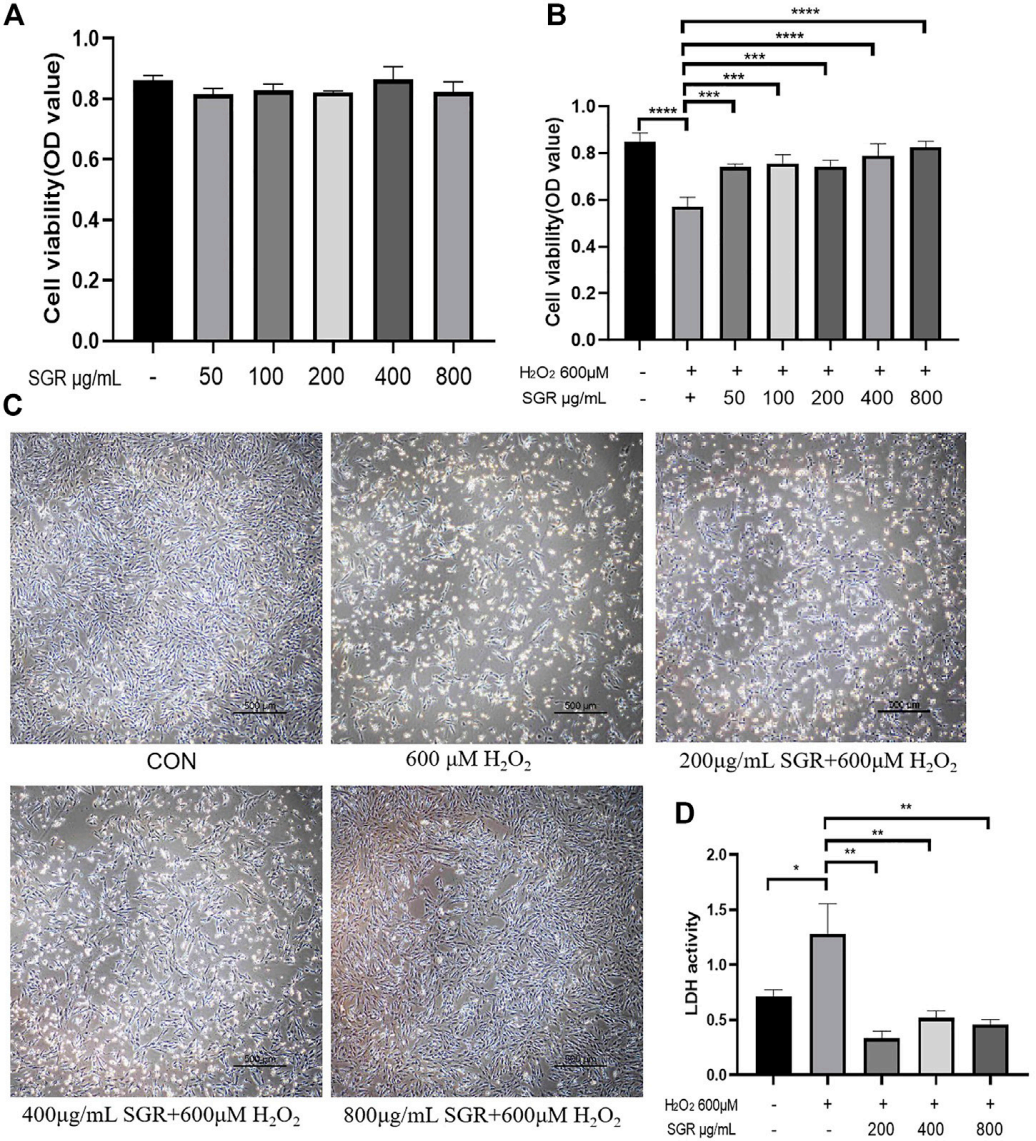
Cytotoxicity and protective effect of different concentrations of SGR on H9c2 cells. **(A)** Cytotoxicity of different concentration of SGR on H9c2 cells; **(B)** protective of SGR on H_2_O_2_ induced cellular damage; **(C)** SGR restored the cellular morphology damage induced by H_2_O_2_; and **(D)** SGR inhibited the secretion of LDH induced by H_2_O_2_ (*p < 0.05, **p < 0.01, and ***p < 0.001), and *Smilax glabra* Roxb protected H9c2 cells from H_2_O_2_-induced injury by reducing reactive oxygen species.

As Figure 4B showed, the cell viability of the H_2_O_2_ group was significantly reduced, but cellular viability increased as the concentration increased in the SGR pretreatment group. MTT results indicated the concentration of SGR range 0–800 μg/ml had no obvious cytotoxicity (Figure 4A). Thus, 200, 400, and 800 μg/ml (with the best protective effect) of SGR were selected for later experiments. As Figure 4C showed, 200, 400, and 800 μg/ml SGR could restore the cellular morphology damage induced by H_2_O_2_. Cells in the control group arranged regularly and tightly, but they became sparsely arranged and round-shaped, and necrotic cells presented as white bright aperture in the H_2_O_2_ group. In the 200, 400, and 800 μg/ml SGR group, white bright aperture gradually decreased, the fusiform of the cell morphology was gradually restored, and the arrangement began to be regular. It was also verified by the result of LDH (Figure 4D), compared with H_2_O_2_ group, 200, 400, and 800 μg/ml SGR all inhibited the secretion of LDH, indicating that the cellular damage induced by H_2_O_2_ was reversed by SGR.

**FIGURE 5:**
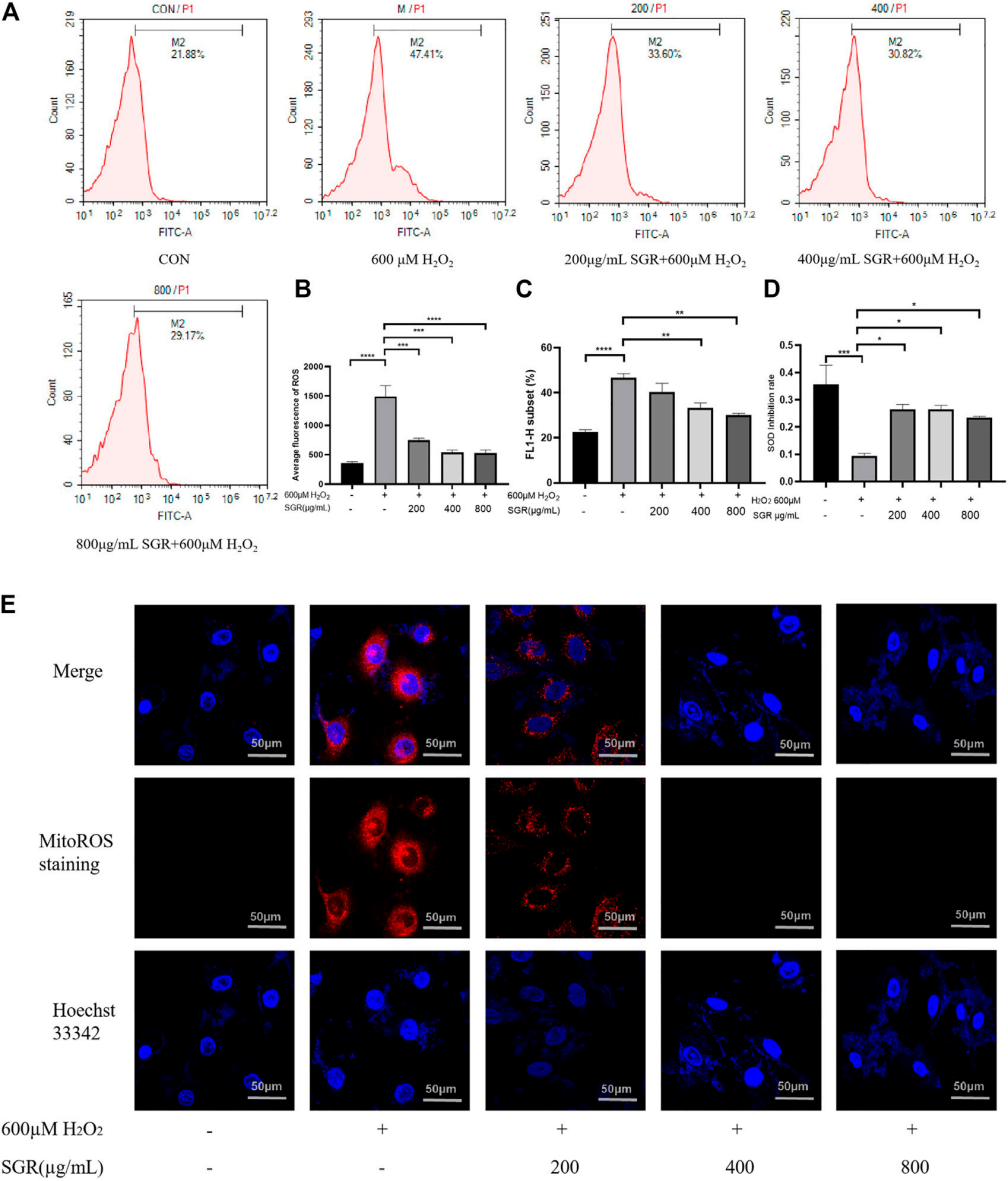
SGR decreased cellular and mitochondrial oxidative stress in H9c2 cells induced by H_2_O_2_. **(A)** The single fluorescence peak of ROS in each group; **(B)** average fluorescence of each group presented the cellular oxidative stress level; **(C)** FL1-H subset presented the proportion of the cells in the same area, containing the cells with increased ROS production; **(D)** SGR increased the SOD inhibition rate induced by H_2_O_2_; and **(E)** fluorescence degree of each group presented the mitochondrial oxidative stress level (×200) (*p < 0.05, **p < 0.01, and ***p < 0.001). *Smilax glabra* Roxb inhibited H_2_O_2_-induced lysosome in H9c2 cardiomyocytes.

Overexpression of ROS damages mitochondrial function, which leads to further oxidative toxicity (Takano et al., 2003). Oxidative injury and mitochondrial dysfunction induce the significant release of intracellular ROS. As shown in Figures 5A–C, H_2_O_2_ exposure increased cellular ROS production reflected by that the fluorescence peak shifted to the right and average fluorescence increased in the H_2_O_2_ group. However, with the pretreatment of SGR, the fluorescence peak began to shift to the left gradually, they all showed significant differences compared with the H_2_O_2_ group. It was verified that SGR increased the expression of SOD, revealing SGR did inhibit the oxidative stress in the H_2_O_2_-induced model (Figure 5D). In addition, SGR treatment also significantly prevented H_2_O_2_-induced mitochondrial ROS production measured by MitoROS staining. Mitochondrial ROS (MitoROS) is the most important source of intracellular ROS. The results to explore the effect of SGR on the MitoROS production showed that H_2_O_2_ induced the elevation of MitoROS production, while SGR pretreatment suppressed this elevation (Figure 5E).

**FIGURE 6:**
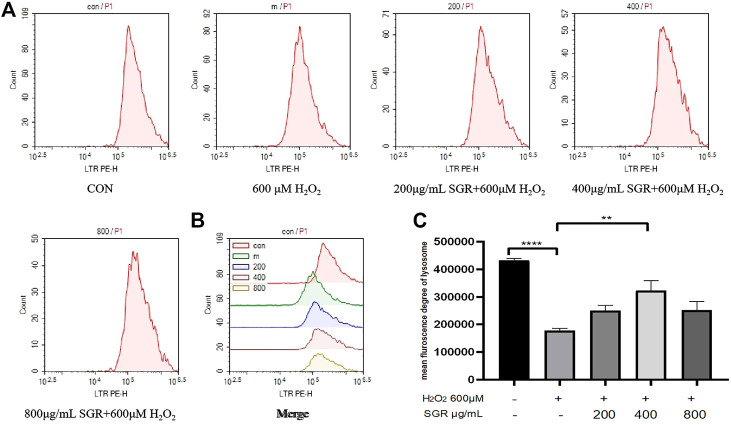
SGR improved lysosomal content in H_2_O_2_-induced damage. **(A)** The single fluorescence peak of lysosomal presented the cellular oxidative stress level in each group; **(B)** merge fluorescence peak of each group presented the cellular oxidative stress level; **(C)** mean fluorescence degree of lysosomal in each group (*p < 0.05, **p < 0.01, and ***p < 0.001). *Smilax glabra* Roxb inhibited H_2_O_2_-induced mitochondrial damage in H9c2 cardiomyocytes.

A lysosome is an important indicator of oxidative stress. Lysosomal dysfunction is associated with autophagy impairment, senescence and Lysosomal disruption is a hallmark of oxidative induced apoptosis (Rajapakse et al., 2017; Tai et al., 2017). In this study, we found that H_2_O_2_ inhibited Lysosome content compared with the control group, but with pretreatment of SGR, Lysosomal content was improved (Figures 6A–C), this result also suggested that SGR protected Lysosomal from H_2_O_2_-induced damage.

**FIGURE 7:**
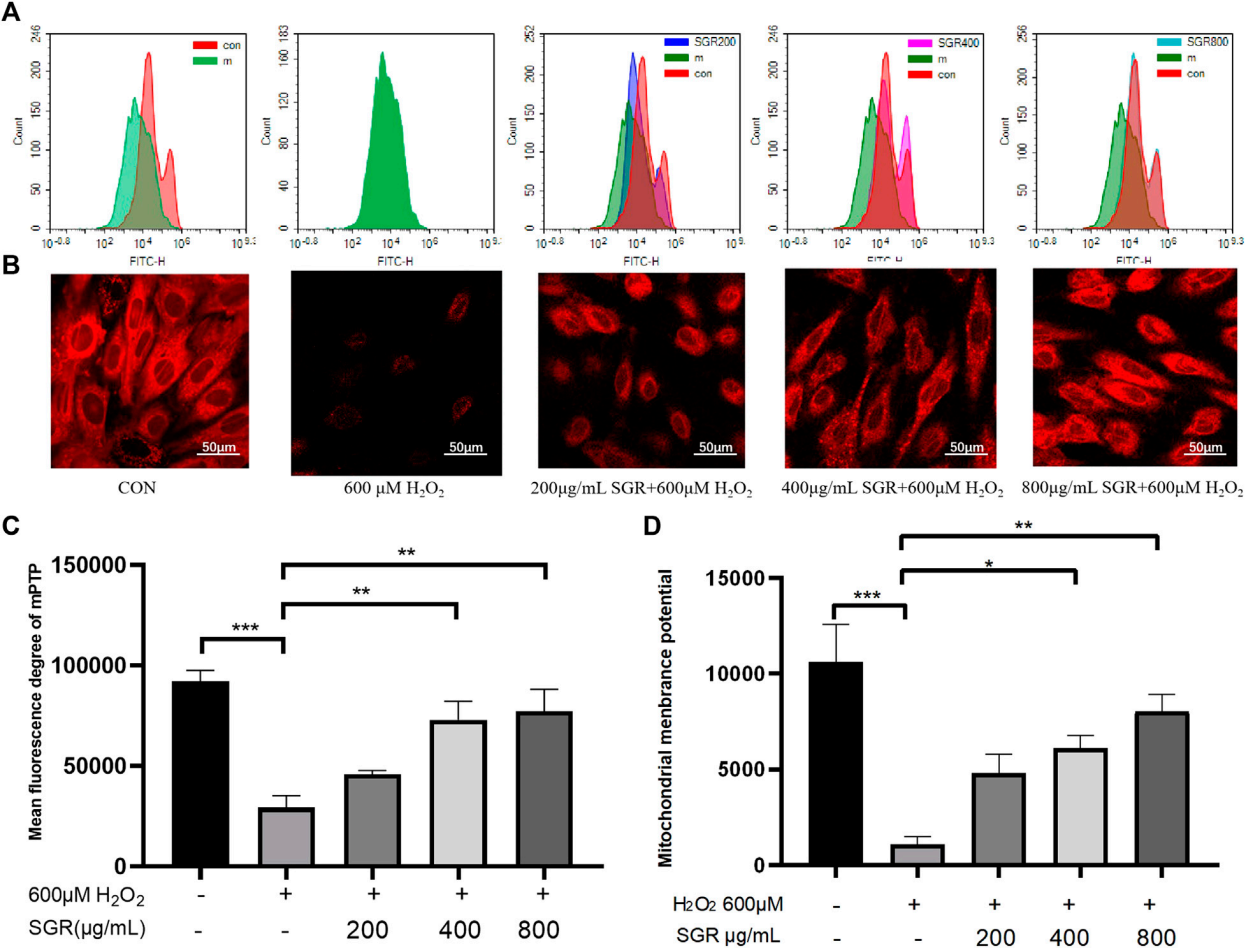
SGR protected mitochondrial damage induced by H_2_O_2_, presenting by mitochondrial membrane potential **(B,D)** and mitochondrial permeability transition pore (mPTP) opening **(A,C)**; *p < 0.05, **p < 0.01, and ***p < 0.001. *Smilax glabra* Roxb reduced oxygen consumption in intact H9c2 cardiomyocytes.

To determine whether SGR protected mitochondria damage in response to H_2_O_2_ injury, we examined H_2_O_2_-induced alterations in mitochondrial membrane potential (ΔΨm) presented by Mito-tracker live-cell tag probe and the opening of mitochondrial permeability transition pore (mPTP). mPTP opening assay results showed that mPTP was opening because of the damage of mitochondria induced by H_2_O_2_, but the excessive opening of mPTP was inhibited in the SGR groups, as Figures 7A,C. In addition, we also found that pretreatment with SGR reduced the ΔΨm depolarization, which was indicated by the increased red fluorescence (Figures 7B,D).

**FIGURE 8:**
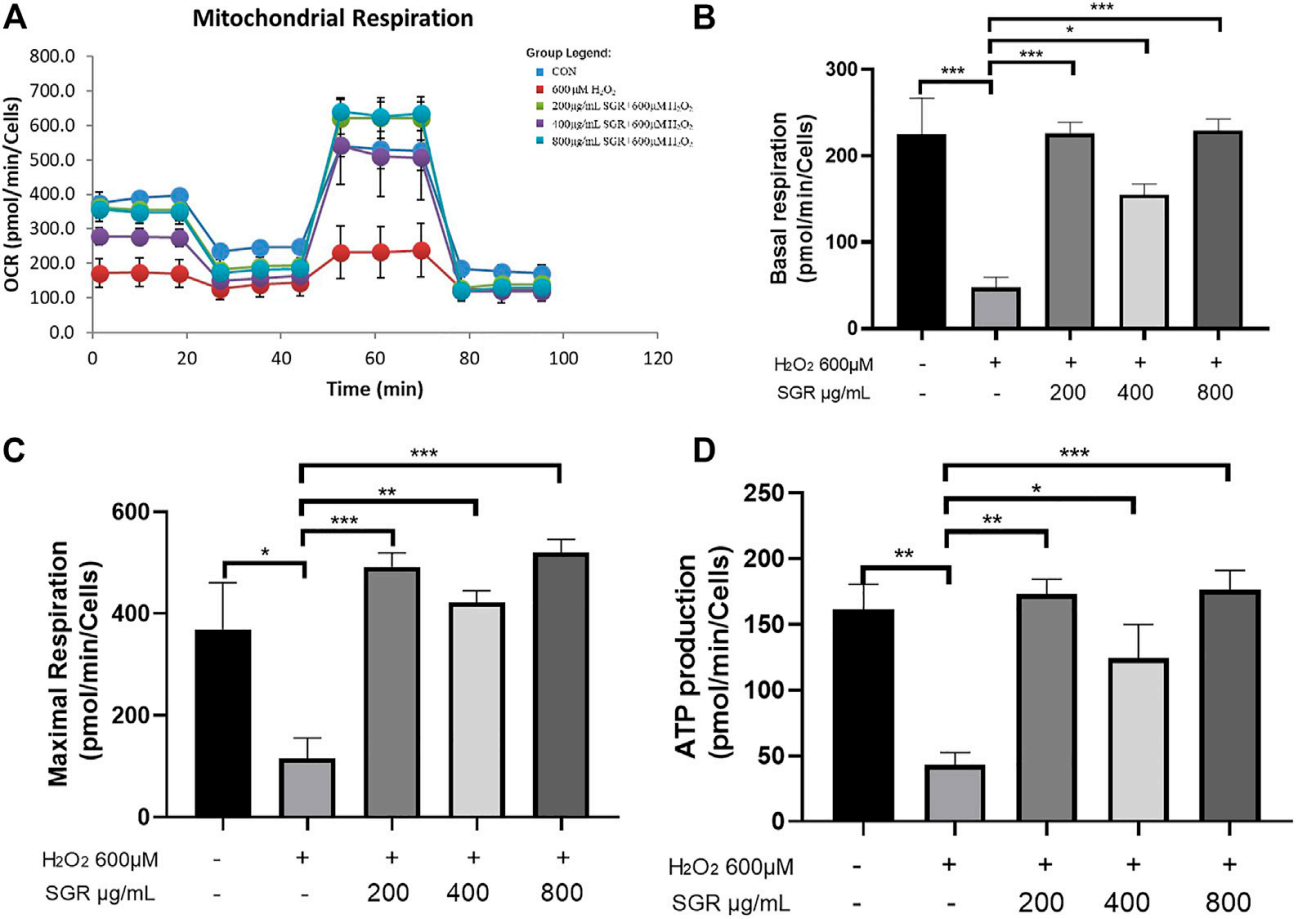
SGR protected mitochondrial respiratory and energy metabolism in H9c2 cells. **(A)** Mitochondrial respiration of oxygen consumption rate (OCR) measured by using an Agilent Seahorse XF metabolic Analyzer, presenting by basal respiration **(B)**, maximum respiration **(C)**, and ATP production **(D)** (*p < 0.05, **p < 0.01, and ***p < 0.001). SGR reduced H_2_O_2_-induced H9C2 cell injury *via* the MAPK signaling pathway.

Figures 8A–D showed that compared with the H_2_O_2_ group, the OCR values of basal respiration, maximum respiration and ATP production of the SGR treatment group increased significantly. It revealed that SGR was capable of protecting mitochondrial respiratory and mitochondrial energy metabolism in rat H9c2 cardiomyocytes against H_2_O_2_-induced mitochondrial dysfunction.

**FIGURE 9:**
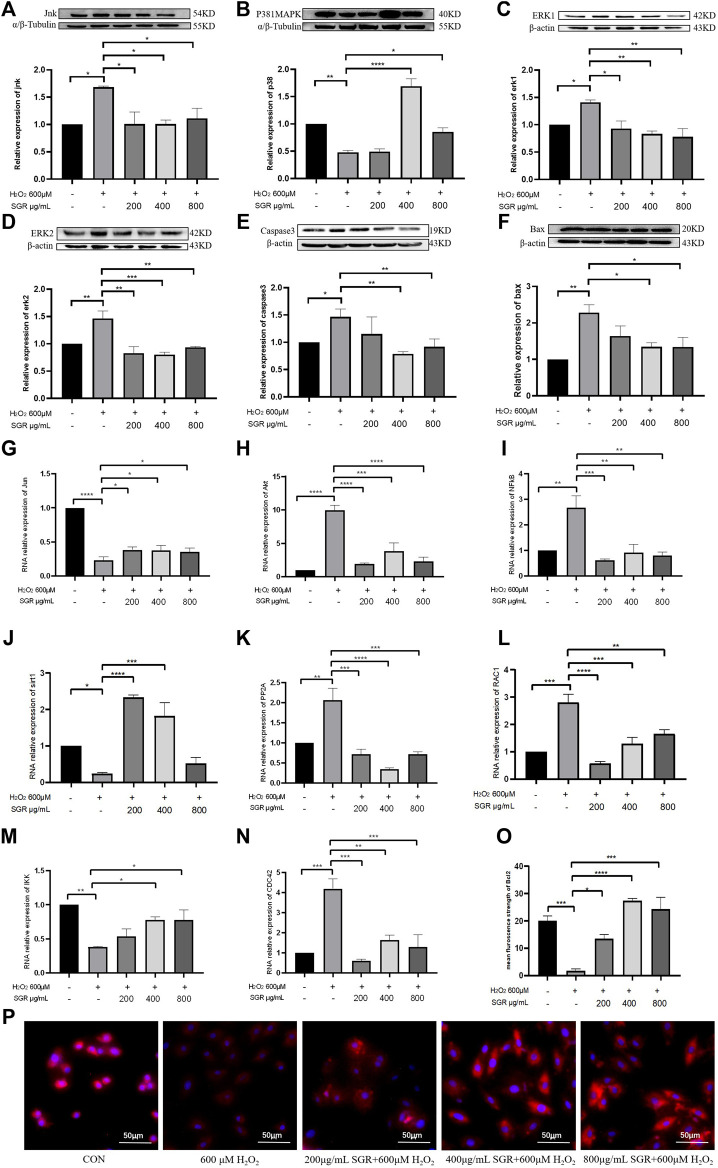
Protein expression and mRNA expression level of p38MAPK pathway modulated by SGR. **(A–F)** Protein expression of P38MAPK, JUN, ERK1, ERK2, Bax, and Caspase3; **(G–N)** mRNA expression level of JUN, AKT, NF-KB, PP2A, CDC42, RAC1, IKK, and Sirt1 in H9c2 cells. **(O)** Mean fluorescence strength of Bcl-2 protein expression detected by immunofluorescence. **(P)** Bcl-2 protein expression revealed by immunofluorescence and imaged by using an inverted fluorescent microscope (*p < 0.05, **p < 0.01, and ***p < 0.001) and identification of hub components contained in SGR.

To investigate the possible mechanism of SGR on H_2_O_2_-induced cellular damage and mitochondrial dysfunction, aiming to verify the pathway predicted by network pharmacological analysis, we explored the SGR modulation on the p38MAPK pathway. It indicated that compared with the H_2_O_2_ group, SGR decreased the mRNA levels of CDC42, PP2A, RAC1, Akt, NFkB (Figures 9N,K,L,H,I), and increased the mRNA levels of IKK, Sirt1, and Jun (Figures 9M,J,K). WB results revealed that H_2_O_2_ increased protein expression of ERK1, ERK2, JNKs, Bax, Caspase3 (Figures 9A,C–F) and decreased p38MAPK and Bcl-2 expression (Figures 9B,O,P), but these were all reversed by SGR pretreatment. All suggested that SGR significantly activated p38MAPK pathways that was inhibited by H_2_O_2_.

**FIGURE 10:**
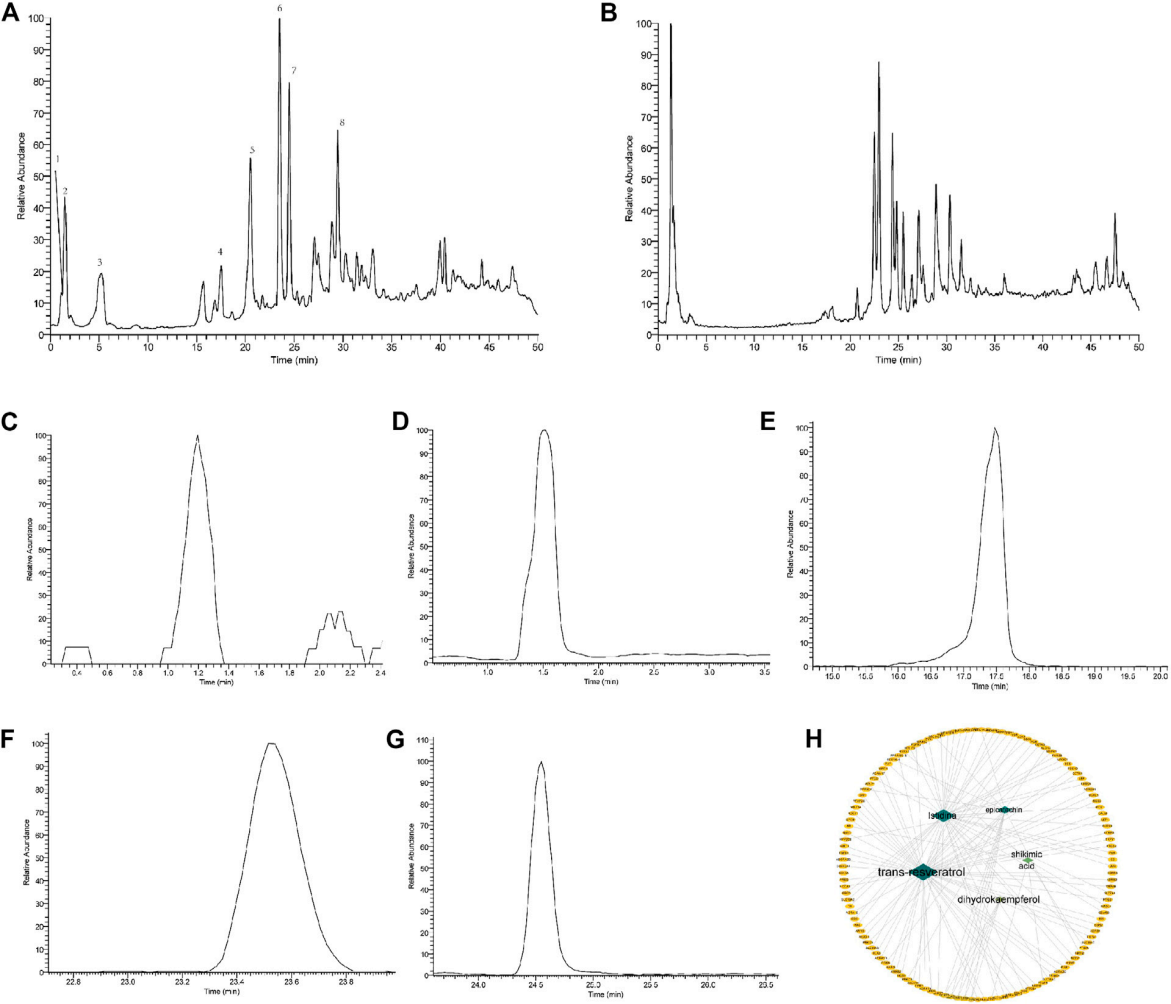
Identification of the components contained in *Smilax glabra* Roxb *via* the UHPLC-LTQ-Orbitrap-MSn method. **(A)** Total ion chromatogram of standards in negative mode; **(B)** total ion chromatogram of SGR in negative mode; **(C)** extracted ion chromatogram of Istidina (m/z: 154.06, RT: 1.20); **(D)** extracted ion chromatogram of shikimic acid (m/z: 173.05, RT: 1.52); **(E)** extracted ion chromatogram of epicatechin (m/z: 289.07, RT: 17.47); **(F)** extracted ion chromatogram of dihydrokaempferol (m/z: 287.06, RT: 23.54); **(G)** extracted ion chromatogram of resveratrol (m/z: 227.07, RT: 24.53); and **(H)** distribution characteristic between identified components of SGR and their predicted targets.

Figures 10A–H showed, extracting masses in negative ionization mode for compounds 5 suggested a molecular formula of C_6_H_9_N_3_O_2_, C_14_H_12_O_3_, C_7_H_10_O_5_, C_15_H_14_O_6_, C_15_H_12_O_6_, with the Molecular Weight of 155.15, 228.24, 174.15, 290.27, 288.25 Da, respectively. By referring to the retention time and secondary fragment ions, Istidina, resveratrol, shikimic acid, epicatechin, and dihydrokaempferol in SGR were accurately identified with the same retention time of 1.20, 24.53, 1.52, 17.47, 23.54 min, the molecular weight of 154.06, 227.07, 173.05, 289.07, 287.06 Da and the same fragment ions.

### Molecular Docking

**FIGURE 11:**
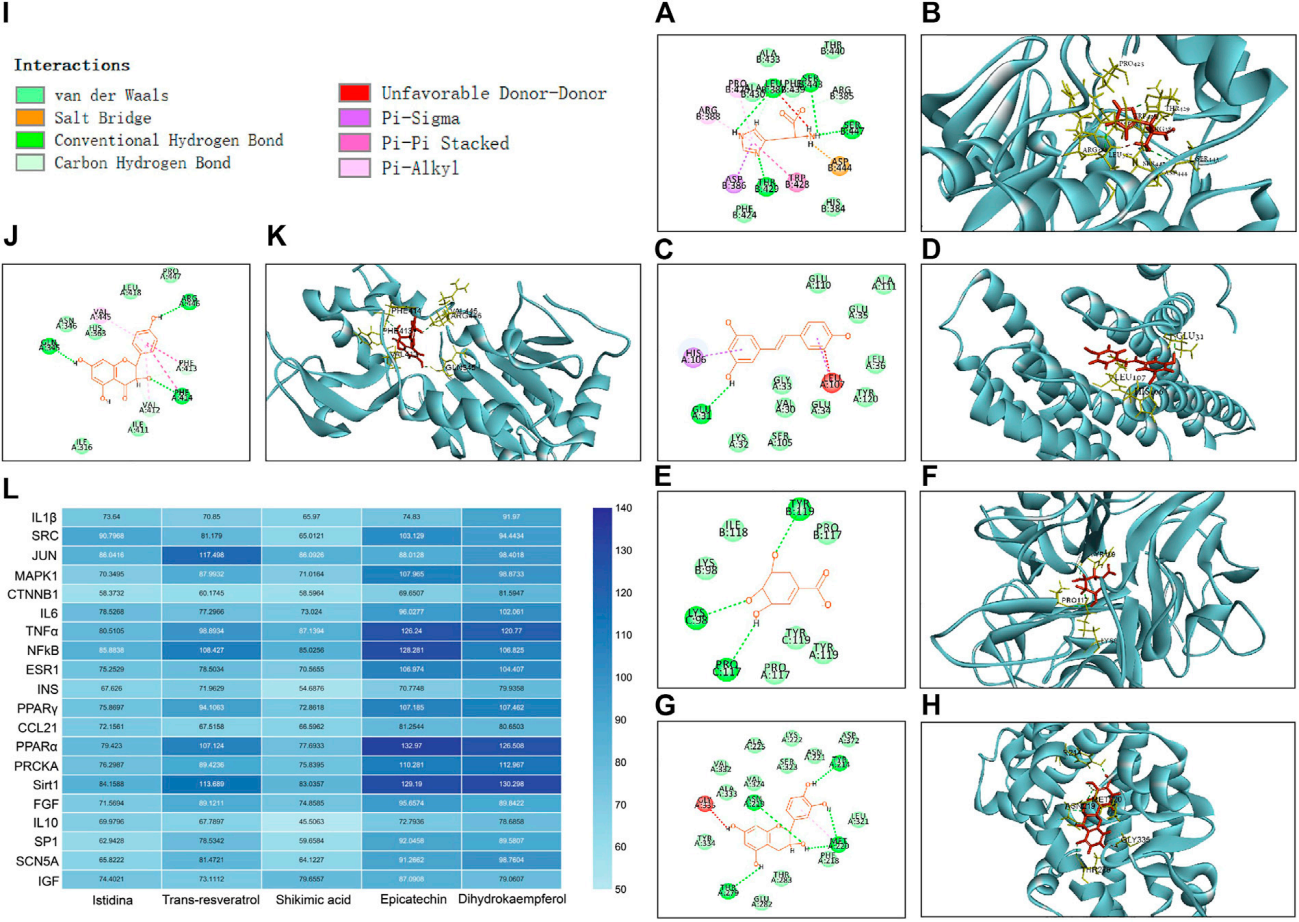
2D and 3D docking structure and docking score of five components docked with their predicted targets. **(A)** 2D docking structure of Istidina docked with SRC; **(B)** 3D docking structure of Istidina docked with SRC; **(C)** 2D docking structure of Trans-resveratrol with JUN; **(D)** 3D docking structure of Trans-resveratrol docked with JUN; **(E)** 2D docking structure of Shikimic acid docked with TNFα; **(F)** 3D docking structure of Shikimic acid docked with TNFα; **(G)** 2D docking structure of epicatechin docked with PPARα; **(H)** 3D docking structure of epicatechin docked with PPARα; **(I)** type of interaction between identified compounds and their docking targets; **(J)** 2D docking structure of dihydrokaempferol docked with Sirt1; **(K)** 3D docking structure of dihydrokaempferol docked with Sirt1; and **(L)** hot map of docking score of the five components docked with their predicted targets.

According to network topology analysis, Istidina and trans-resveratrol were identified as the key active components in SGR. Shikimic acid (6 in degree), epicatechin (7 in degree), and dihydrokaempferol (5 in degree) were also identified by the UHPLC-LTQ-Orbitrap-MSn method. SRC, JUN, CTNNB1, MAPK1, IL6, TNF, NFKB1, IL1B, INS, ESR1, PPARγ, CCL21, PPARA, IGF1, SIRT1, FGF2, IL10, PRKCA, SP1, and SCN5A were the key targets of SGR, and they were docked with the identified component of SGR to clarify the interaction relationships between the key targets and SGR active compound. The results showed that Istidina, Trans-resveratrol, Shikimic acid, Epicatechin, and Dihydrokaempferol had higher docking scores with SRC, JUN, TNF, PPARα, and Sirt1, respectively (Figure 11L), indicating these 5 drugs may have anti-heart failure effect by targeting SRC, JUN, TNF, PPARα, and Sirt1, respectively. Figures 11A–K showed the 2D and 3D docking structure of 5 components docked with their predicted targets.

## Discussion

Cardiovascular diseases are still a challenge for physicians and researchers in the world (Zhang et al., 2017), and their high morbidity and mortality have become a growing public health concern (Tsutsui et al., 2011). Over the past decades, clinical and experimental studies have provided substantial evidence that oxidative stress is defined as the excess production of reactive oxygen species (ROS), leading to cellular dysfunction (Tsutsui et al., 2011) and mitochondrial dysfunction, which further upregulates ROS production and promotes the development of heart failure (Zhang et al., 2021). Mitochondria is an important organelle for ROS production in cardiomyocytes, regulation of ROS and mitochondrial metabolism in HF appears to present benefits for HF in both animal models and clinical populations (Kiyuna et al., 2018).

In this study, network pharmacological analysis results predicted that the important BP of SGR participated in the treatment of HF included regulation of DNA and RNA transcription, cell proliferation, oxidation-reduction process, apoptotic process, ERK1, and ERK2 cascade, and MAPK cascade, etc. Oxidative stress results in DNA damage and worsens mitochondrial function, leading to organ dysfunction and even aggravating the development of heart failure (Wang C. et al., 2020). RNA and DNA transcription or translation, and cardiomyocyte proliferation are critical for myocardial growth, they both participate in promoting cardiac regeneration in the condition of cardiac injury in response to externally induced hypertrophy (Gao and Wang, 2020) and reversing cardiac remodeling (Wang Y. et al., 2020). The ERK cascade is capable of regulating cellular damage under cardiac stress when ERK is exposed to external stimuli, resulting in important irreversible cardiac damage, such as heart failure (Gallo et al., 2019). In addition, the associated targets enriched in CC mainly included mitochondrion and endoplasmic reticulum. Also, MF mainly included protein binding, ATP binding, enzyme binding, and receptor binding. During the early stages of oxidative stress, calcium is excreted from the endoplasmic reticulum and is recruited by mitochondria through protein binding, enzyme binding, or receptor binding (Hove-Madsen and Bers, 1992). It increases calcium concentrations and further increases metabolic activity and ROS production in mitochondria. Increasing ROS in mitochondria triggers calcium release from the endoplasmic reticulum and causes calcium release channels or ATP channel activation (Paggio et al., 2019) in the endoplasmic reticulum membrane to send feedback signals (Zeeshan et al., 2016). The imbalance of calcium in the endoplasmic reticulum can lead to cardiac hypertrophy, and severe or prolonged endoplasmic reticulum and mitochondrial stress can lead to cardiomyocyte apoptosis and even result in chronic heart failure (Dickhout et al., 2011). Thus, it was predicted that SGR protected the failing heart through an oxidation-reduction process, anti-apoptotic, regulating ERK1 and ERK2 cascade and MAPK cascade by binding protein, ATP, enzyme, or receptor in endoplasmic reticulum or mitochondria.

In verification, we found that SGR did protect cardiomyocyte viability from H_2_O_2_-induced damage. It restored cardiomyocyte morphology injury and inhibited LDH excretion. SGR inhibited cellular oxidative by increasing the level of SOD, improving lysosomal dysfunction, and reducing intracellular ROS levels. In addition, SGR protected mitochondrial function, it protected mitochondrial membrane potential, inhibited opening of mPTP, reduced mitochondrial ROS content, and restored mitochondrial respiration function. These results demonstrated that SGR did effectively inhibit cardiomyocyte oxidative damage, promote cardiomyocyte antioxidant capacity and improve mitochondrial function.

Pathway enrichment analysis results suggested that the mechanisms mainly included MAPK signaling pathway, Calcium signaling pathway, Dilated cardiomyopathy, Hypertrophic cardiomyopathy (HCM), and PPAR signaling pathway, etc. MAPK signaling pathway is an important pathway involved in cellular proliferation and migration, regulating myocyte contractility and cell death in cardiomyocytes (Wang, 2007; Back et al., 2015). ERK and JNK are important molecules of the MAPK pathway (Hernandez et al., 2011). ERK is involved in hereditary cardiomyopathy and severe cardiac hypertrophy (Gallo et al., 2019, Sa et al., 2008). JNKs are activated in response to a variety of stress stimuli, such as oxidative stress and cardiac I/R injury, etc. (Shvedova et al., 2018). JNK contributes to the enhancement of oxidative damage and apoptosis of cardiomyocytes, (Li M. et al., 2019). Bcl-2 family is the downstream pathway of JNKs. The balance between the apoptotic protein Bax and the anti-apoptotic protein Bcl-2 is critical for the cleavage and activation of caspase-3. In this study, network pharmacological analysis predicted MAPK pathway was the underlying mechanism of SGR in treating HF. In our verification, SGR activated MAP kinase (MAPK) signal pathway which was inhibited by H_2_O_2_ induction. SGR decreased the mRNA expression of AKT, PP2A, NF-KB, PP2A, RAC1, CDC42, and the protein expression of ERK1, ERK2, JNK, Bax, Caspase3. SGR also increased the mRNA expression of IKK, Jun, Sirt1, and the protein expression of p38MAPK and Bcl-2. Thus, SGR exerted protection on H9c2 cells by activating p38MAPKs signaling.

UHPLC-LTQ-Orbitrap-MSn methods identified that Istidina, resveratrol, shikimic acid, epicatechin, and dihydrokaempferol were contained in SGR, of which Istidina was predicted as active compound of SGR by network pharmacological analysis. Studies have shown that Istidina has cardioprotective effects *via* reducing the level of triglyceride in the heart of diabetic rats, reducing myocardial mitochondrial damage caused by cerebral ischemia, and inhibiting reactive oxygen species to prevent reperfusion in isolated hearts after ischemia (Lee et al., 2005; Farshid et al., 2014). Also, with the combination treatment with β-alanine, Istidina enhanced the benefits of exercise training in rats with heart failure (Stefani et al., 2020). However, the research of trans-resveratrol on cardiovascular protection is still rare. Other compounds in SGR also have an anti-oxidative effect, including that Shikimic acid could reduce the accumulation of ROS to modulate oxidative stress and apoptosis in Kidneys, and Shikimic acid could reverse H_2_O_2_-induced cellular oxidative damage *via* JNK/Bax pathway to reduce cellular damage (Manna et al., 2014; Rabelo et al., 2015; Lee et al., 2020; Martinez-Gutierrez et al., 2021). Epicatechin and its metabolites could protect against oxidative stress *via* a direct antioxidant effect in the cardiovascular system (Ruijters et al., 2013), gastrointestinal system (Li Y. et al., 2019), and respiratory system (Shariati et al., 2019). Epicatechin-enriched cocoa could alter mitochondrial structure and biogenesis in heart failure patients (Taub et al., 2012). Also, it was reported that Dihydrokaempferol was a flavonoid isolated from traditional Chinese medicine with high antioxidant and anti-inflammatory properties (Zhang et al., 2021). Molecular docking further indicated these 5 identified compounds may exert anti-HF effect by targeting SRC, JUN, TNFα, PPARα, and Sirt1, respectively, but the study on the relationship between identified compounds and their targeted protein is still lacking; thus, we draw a hypothesis that Istidina may be possible anti-HF agent by targeting SRC, which required our further verification in future.

In conclusion, SGR has a protective effect on HF through cellular and mitochondrial protection *via* multi-compounds and multi-targets, its mechanism is involved in activating the p38 MAPK pathway, and Istidina may be possible anti-HF agents by targeting SRC.

## Data Availability

The original contributions presented in the study are included in the article/Supplementary Material, further inquiries can be directed to the corresponding authors.
