# Supplementary figures and images for: Mechanism and Protective Effect of Smilax glabra Roxb on the Treatment of Heart Failure via Network Pharmacology Analysis and Vitro Verification
Source: Front Pharmacol. 2022 May 23;13:868680. doi: 10.3389/fphar.2022.868680 (PMC9169610; doi:10.3389/fphar.2022.868680)

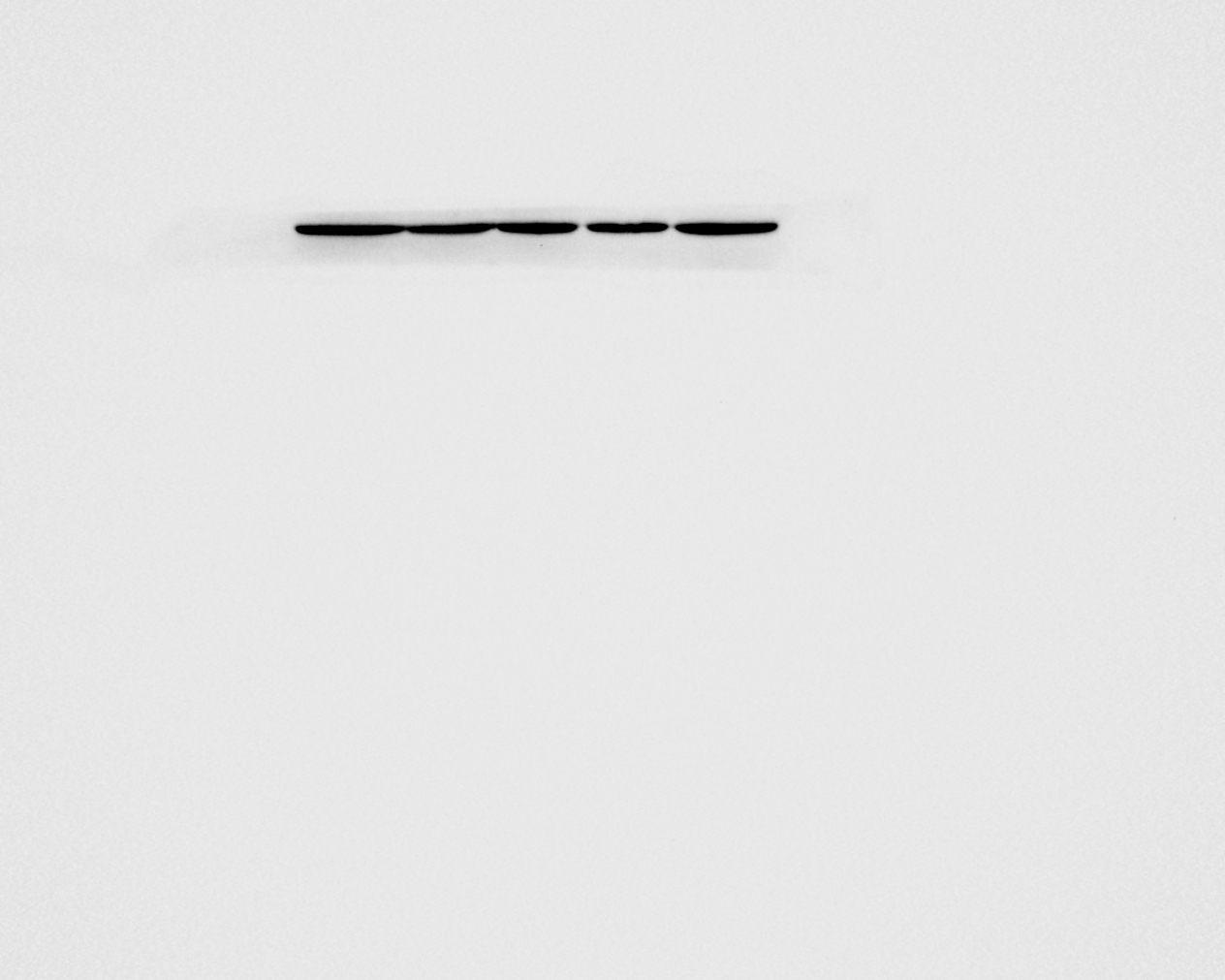

Supplement: Supplementary file 1 [file DataSheet3.ZIP › Weatern blot raw data1/bax/actin 2.jpg]

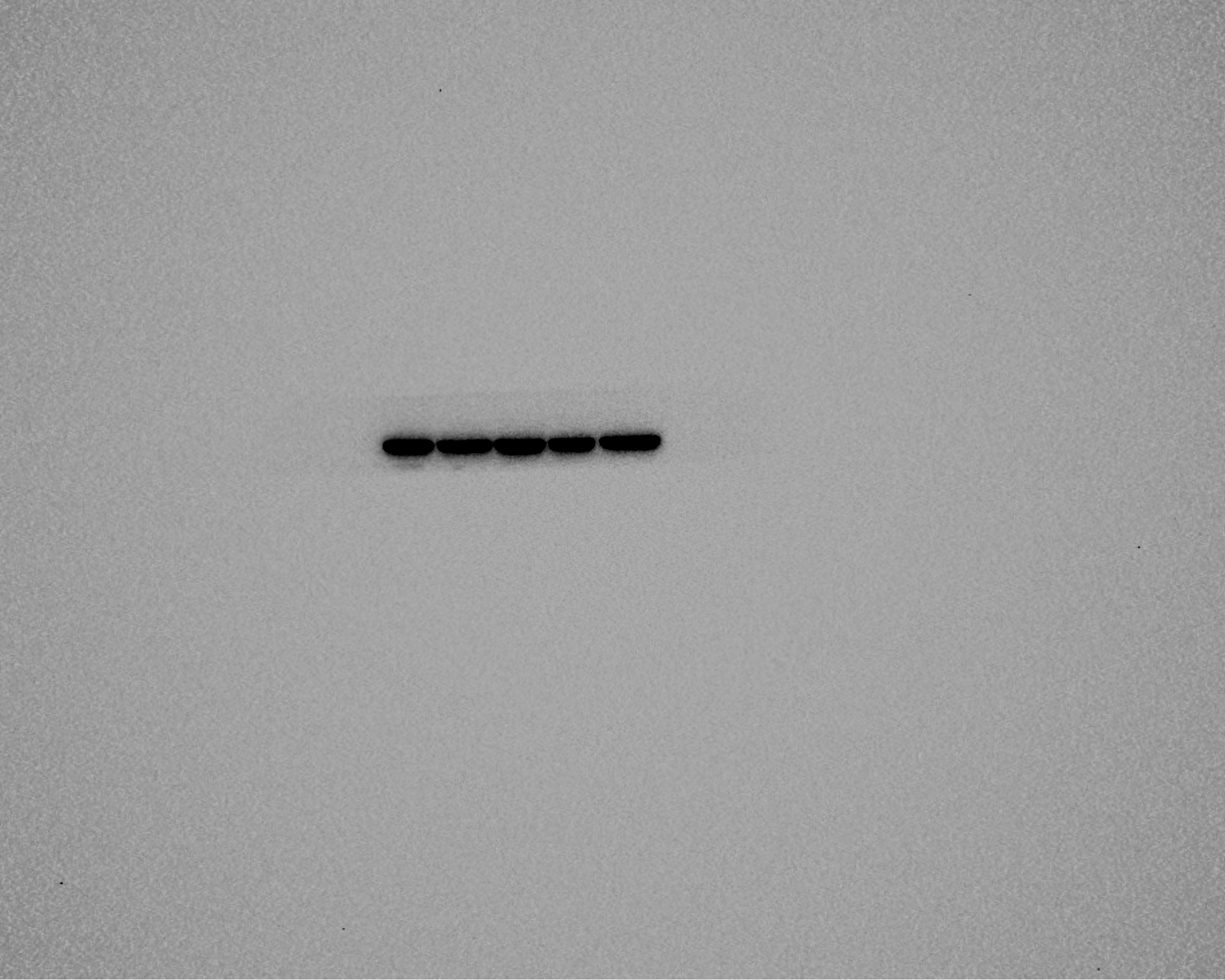

Supplement: Supplementary file 1 [file DataSheet3.ZIP › Weatern blot raw data1/bax/actin3.jpg]

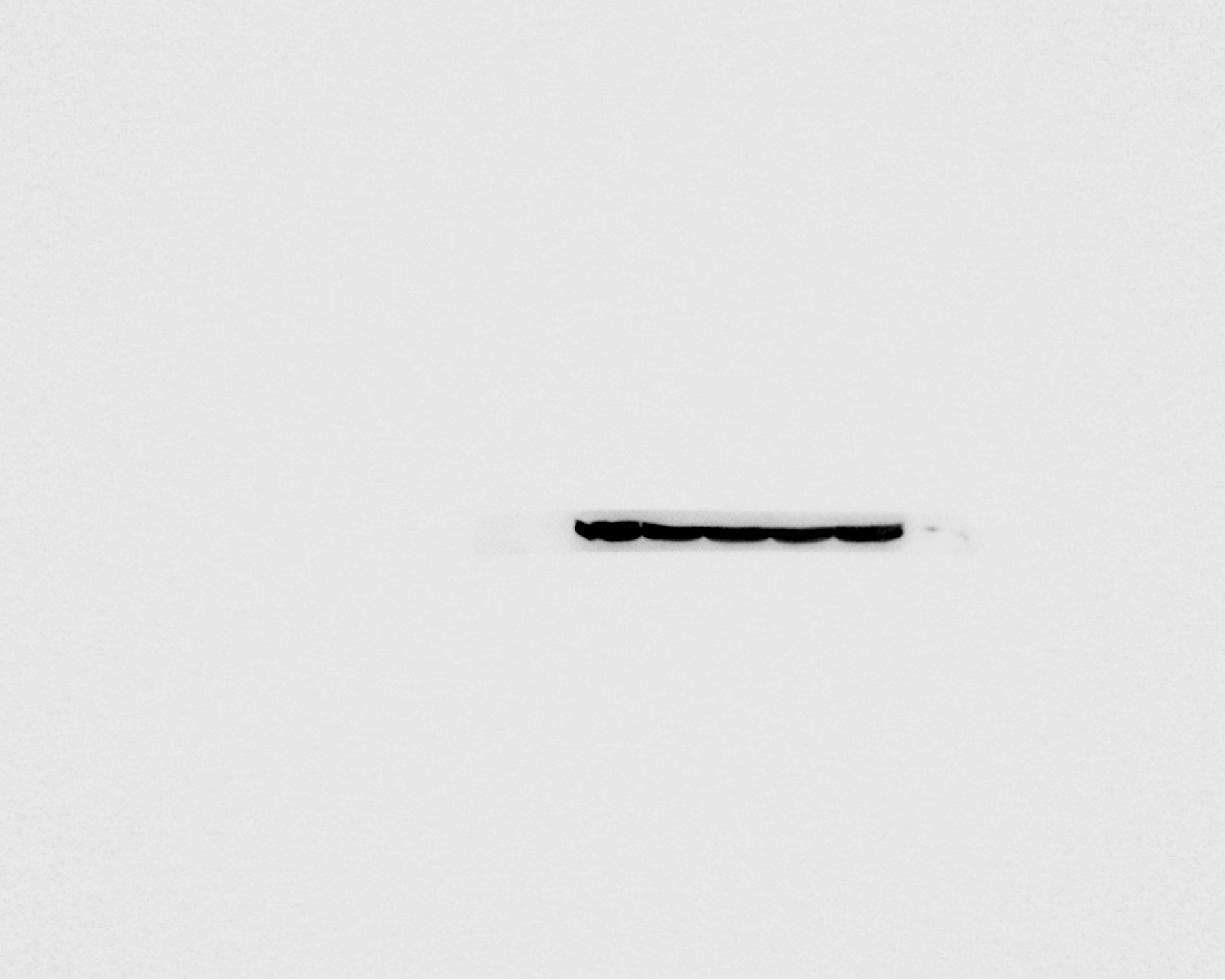

Supplement: Supplementary file 1 [file DataSheet3.ZIP › Weatern blot raw data1/bax/actin_1.jpg]

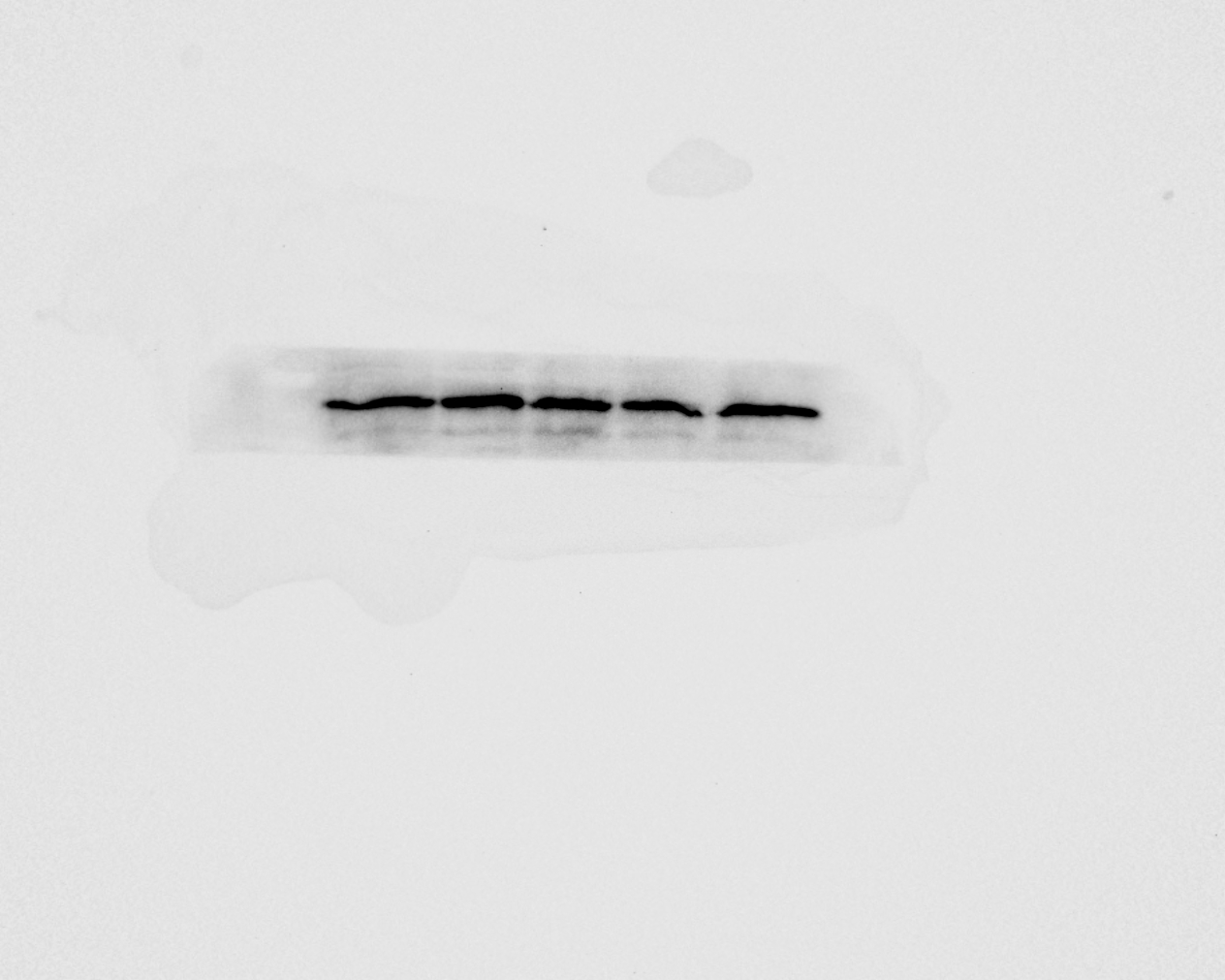

Supplement: Supplementary file 1 [file DataSheet3.ZIP › Weatern blot raw data1/bax/bax2.jpg]

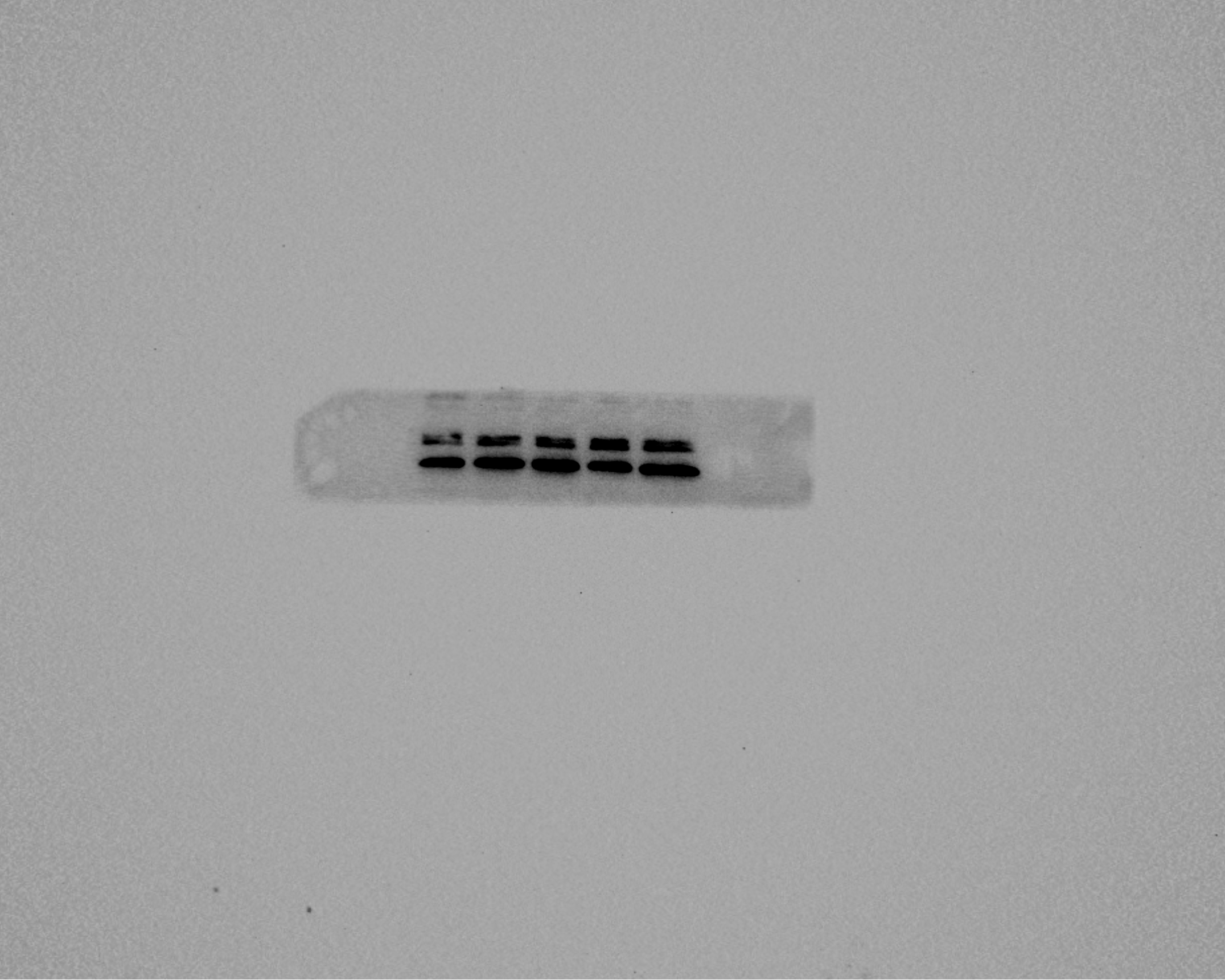

Supplement: Supplementary file 1 [file DataSheet3.ZIP › Weatern blot raw data1/bax/bax3.jpg]

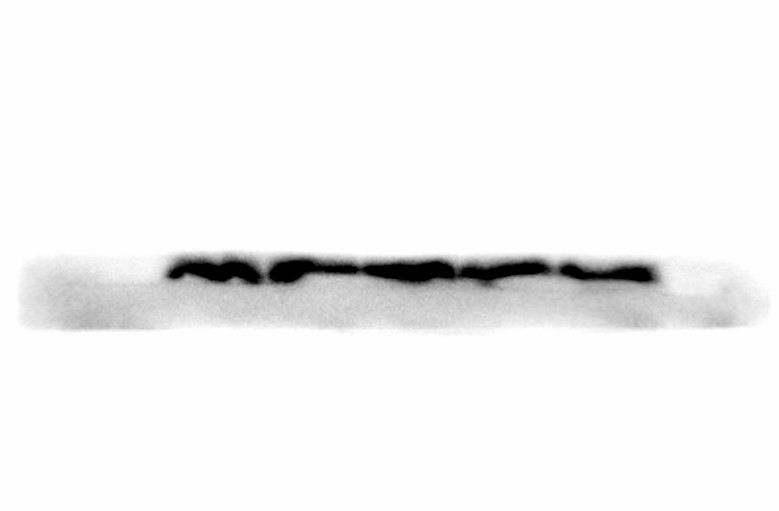

Supplement: Supplementary file 1 [file DataSheet3.ZIP › Weatern blot raw data1/bax/bax_1.jpg]

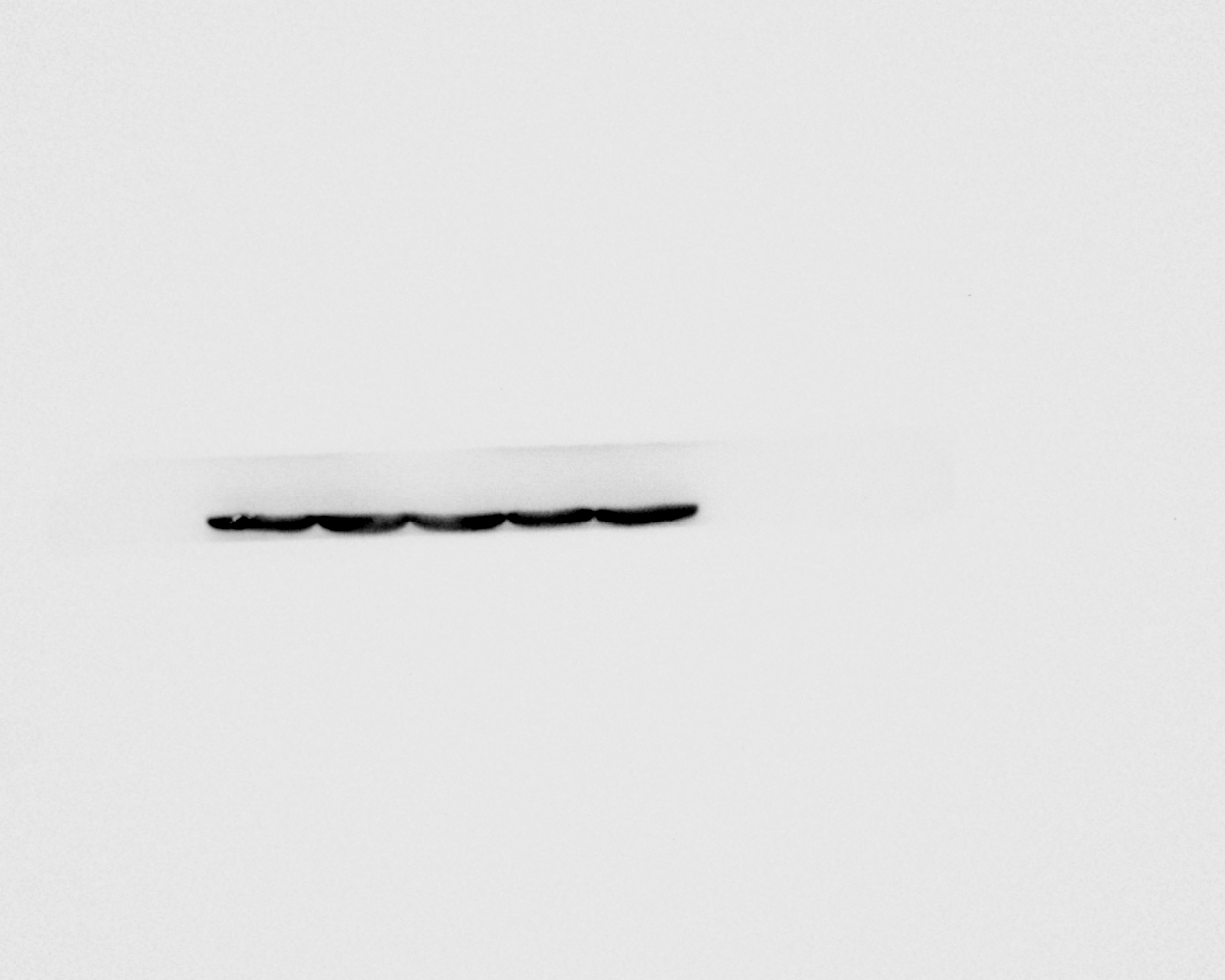

Supplement: Supplementary file 1 [file DataSheet3.ZIP › Weatern blot raw data1/caspase3/actin 1.jpg]

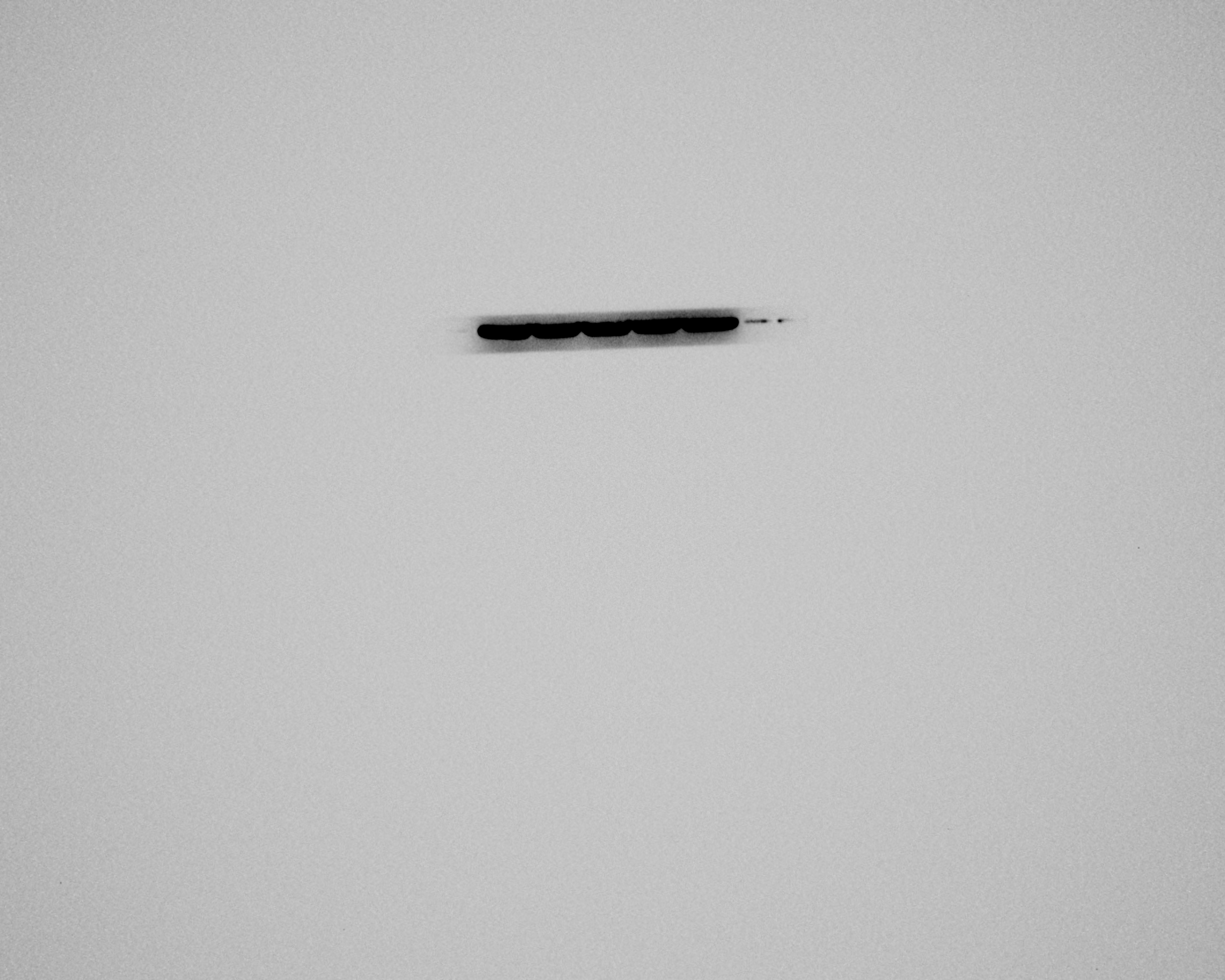

Supplement: Supplementary file 1 [file DataSheet3.ZIP › Weatern blot raw data1/caspase3/actin 3.jpg]

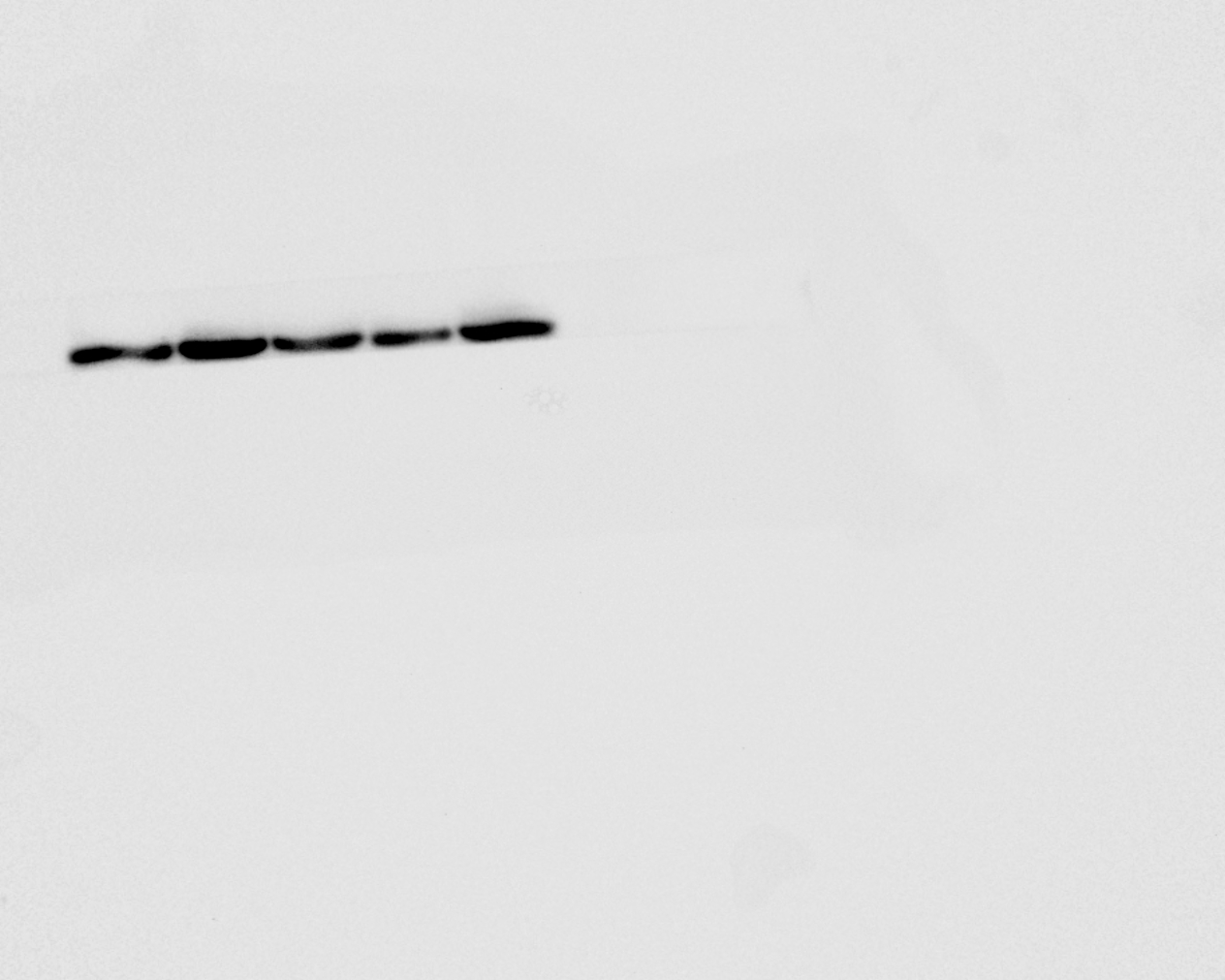

Supplement: Supplementary file 1 [file DataSheet3.ZIP › Weatern blot raw data1/caspase3/cas3 1.jpg]

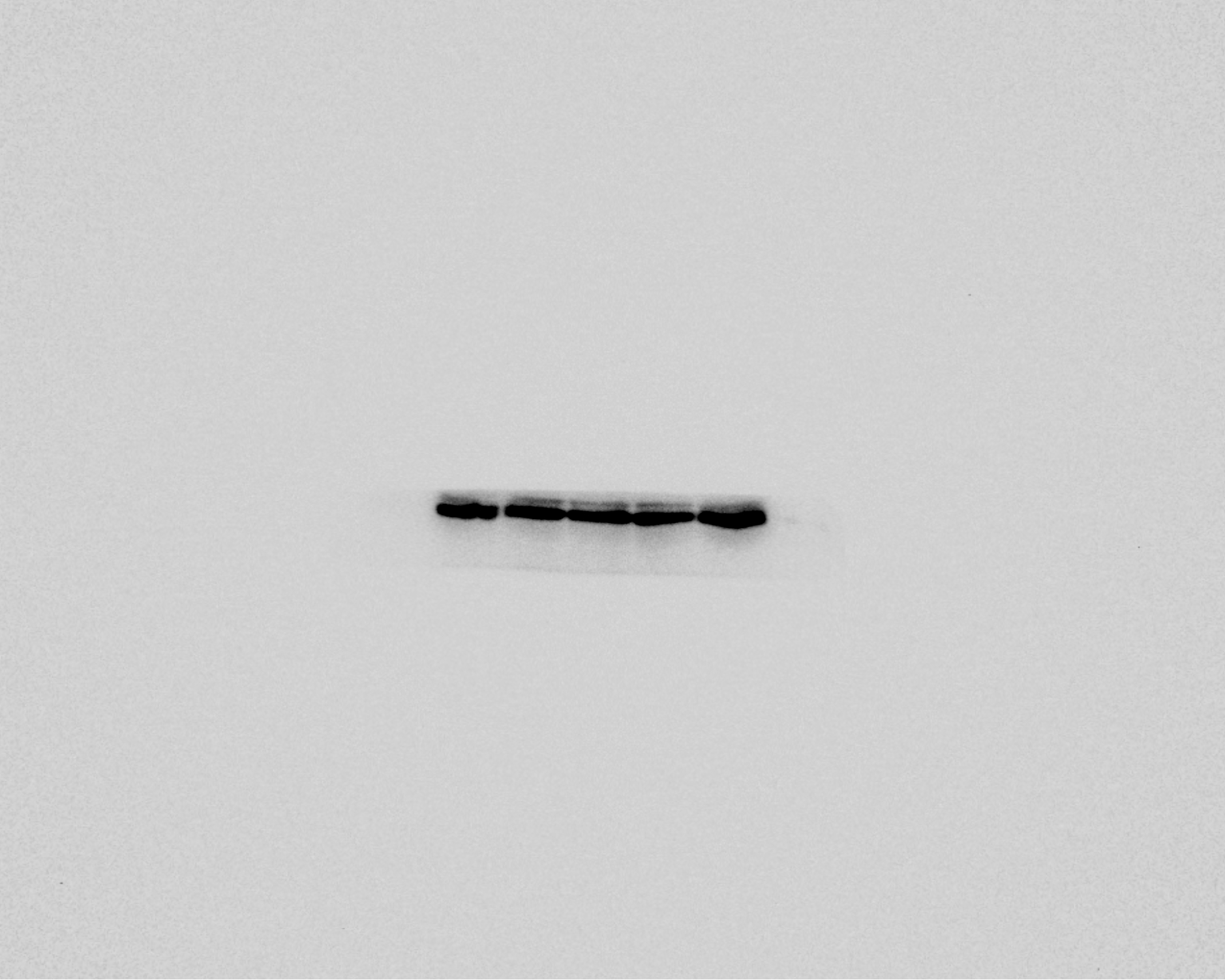

Supplement: Supplementary file 1 [file DataSheet3.ZIP › Weatern blot raw data1/caspase3/cas3 3.jpg]

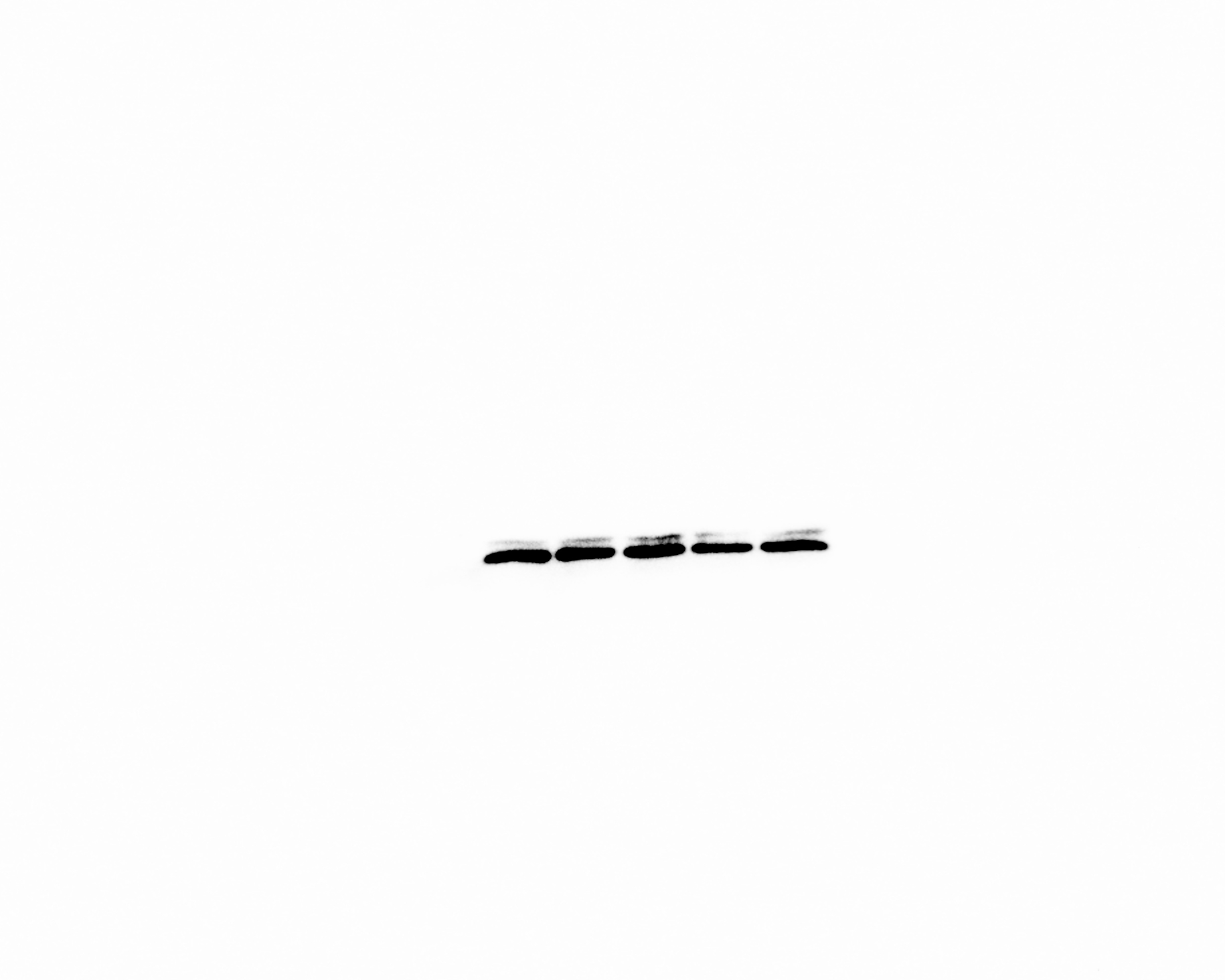

Supplement: Supplementary file 1 [file DataSheet3.ZIP › Weatern blot raw data1/caspase3/cas3_2.jpg]

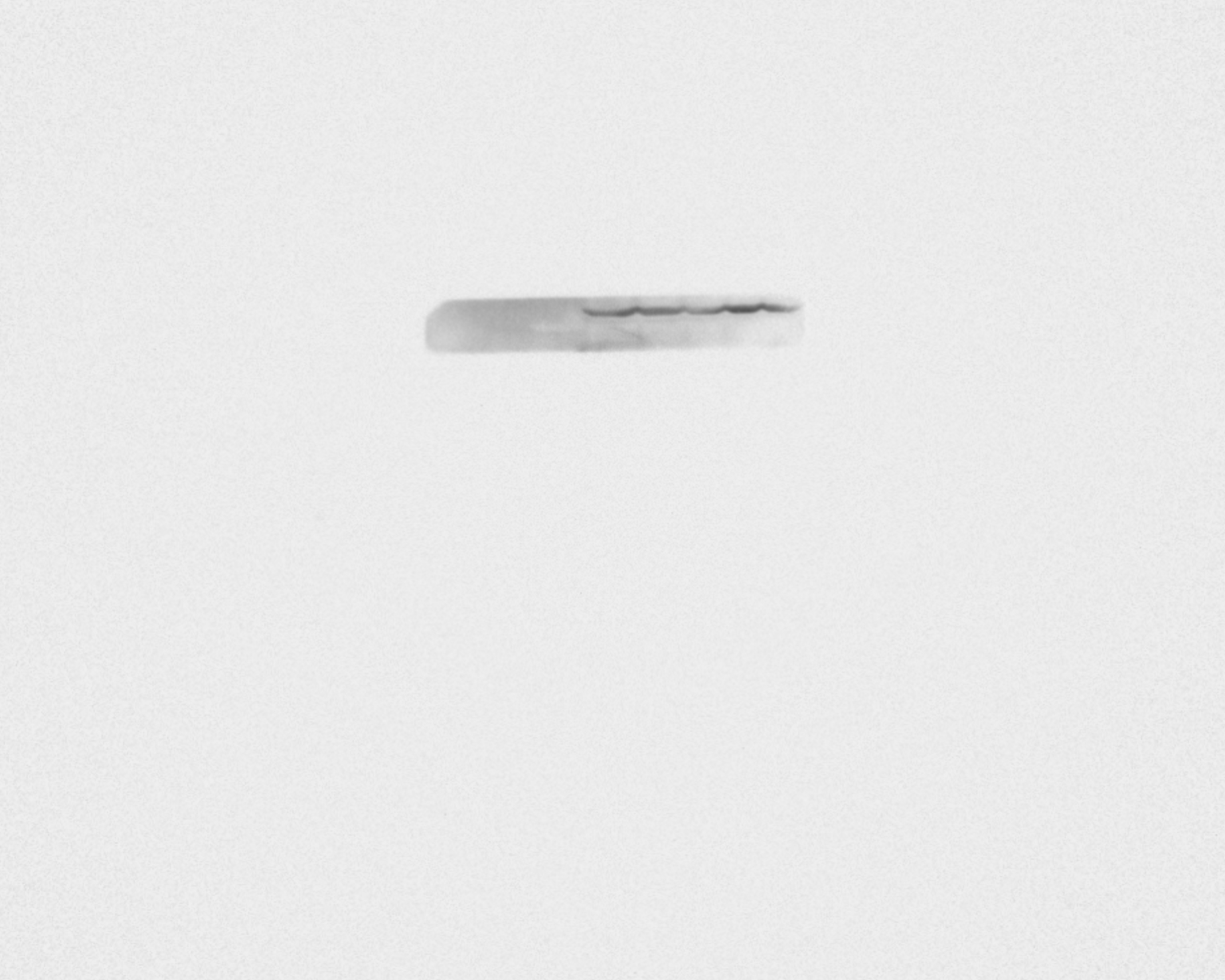

Supplement: Supplementary file 11 [file DataSheet2.ZIP › Weatern blot raw data 2/erk1/actin 3(Chemiluminescence).jpg]

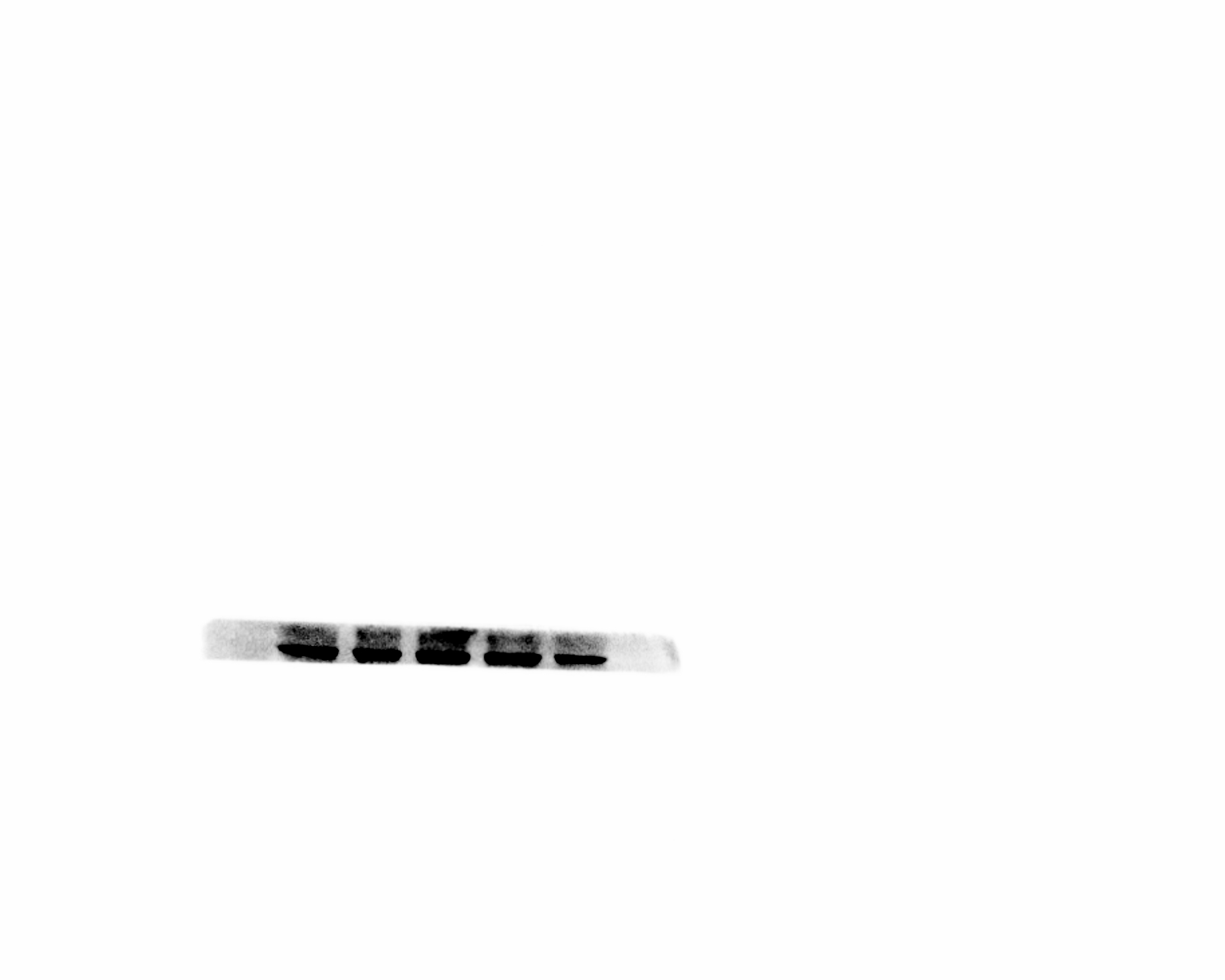

Supplement: Supplementary file 11 [file DataSheet2.ZIP › Weatern blot raw data 2/erk1/actin1.jpg]

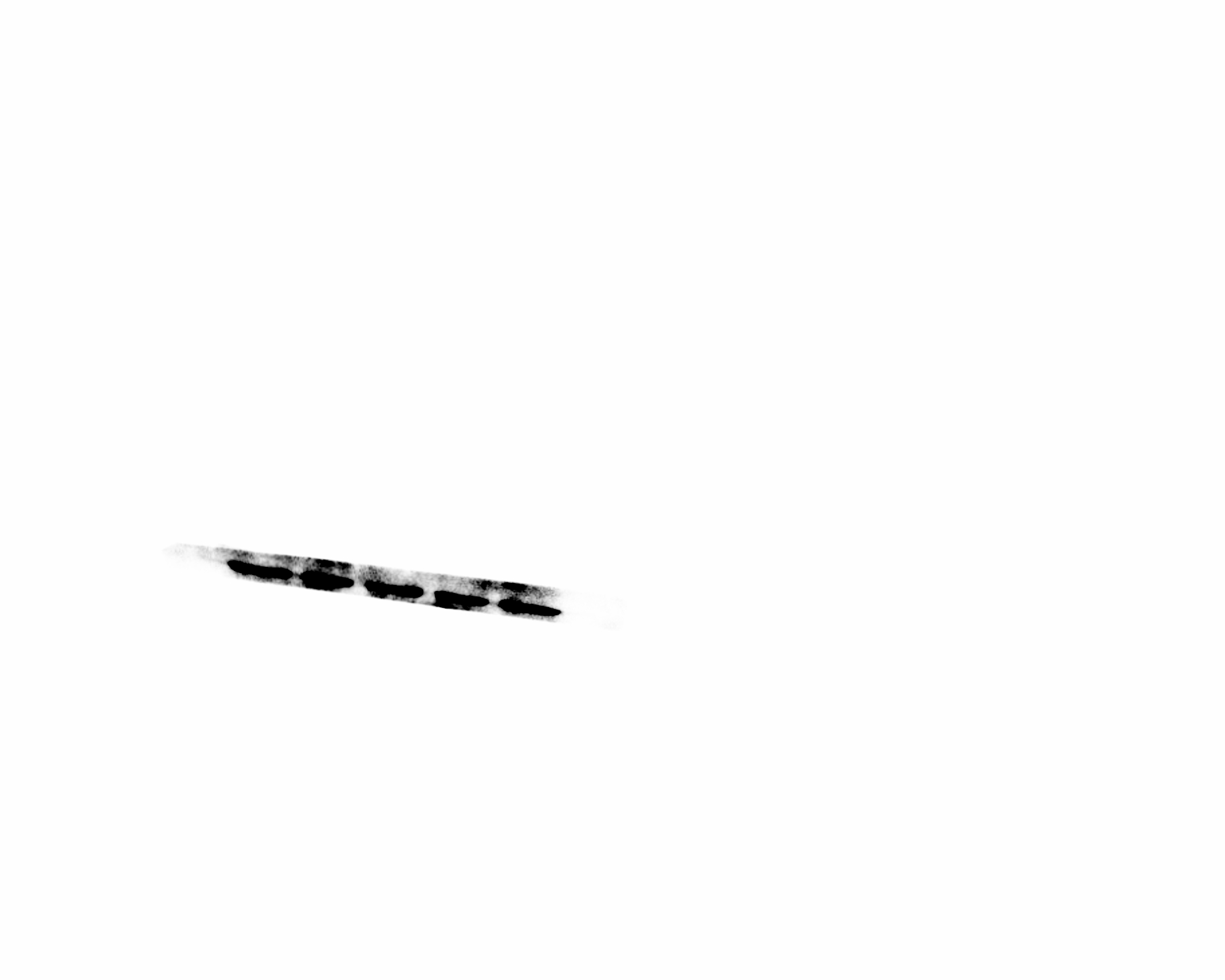

Supplement: Supplementary file 11 [file DataSheet2.ZIP › Weatern blot raw data 2/erk1/actin2.jpg]

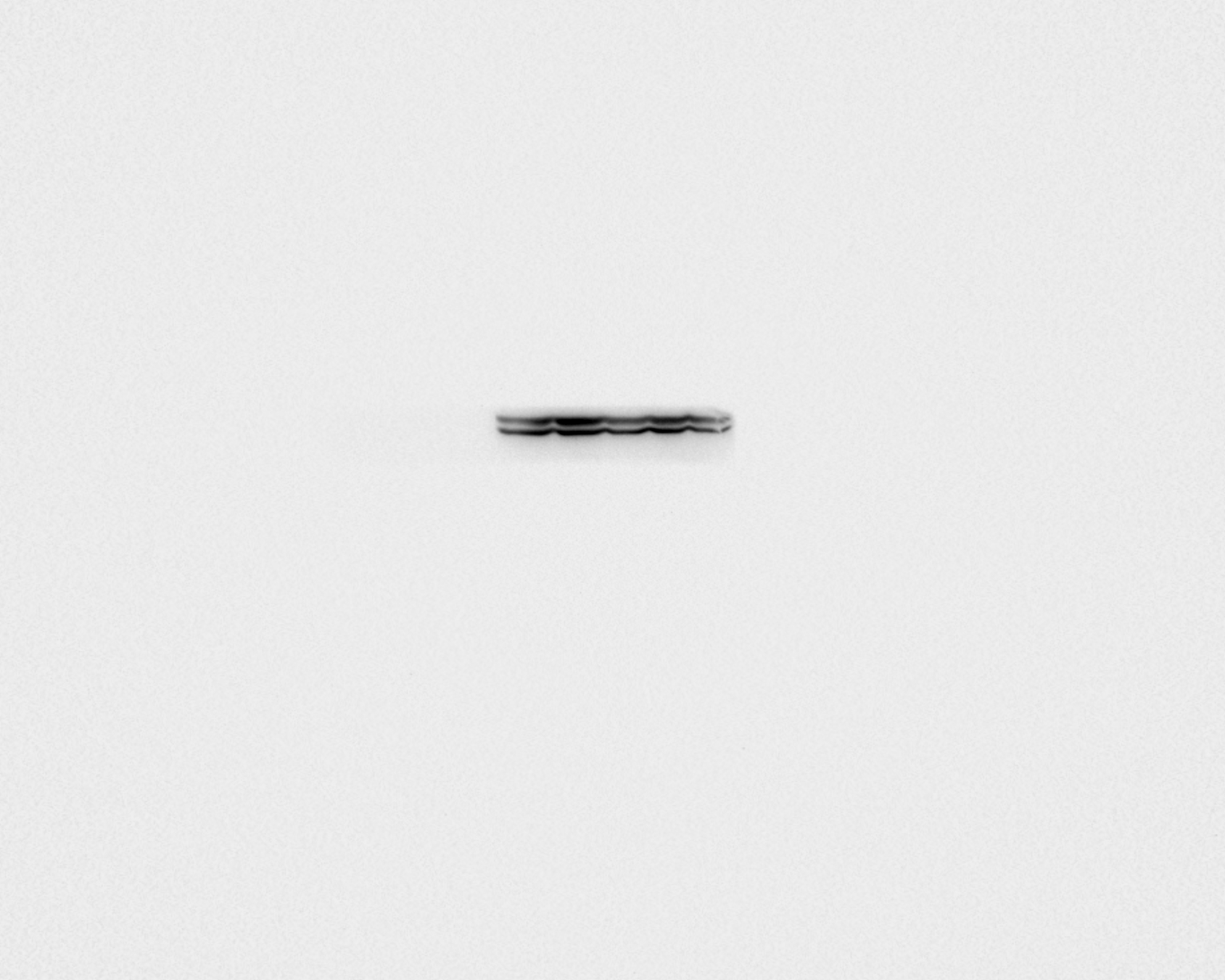

Supplement: Supplementary file 11 [file DataSheet2.ZIP › Weatern blot raw data 2/erk1/erk 3.jpg]

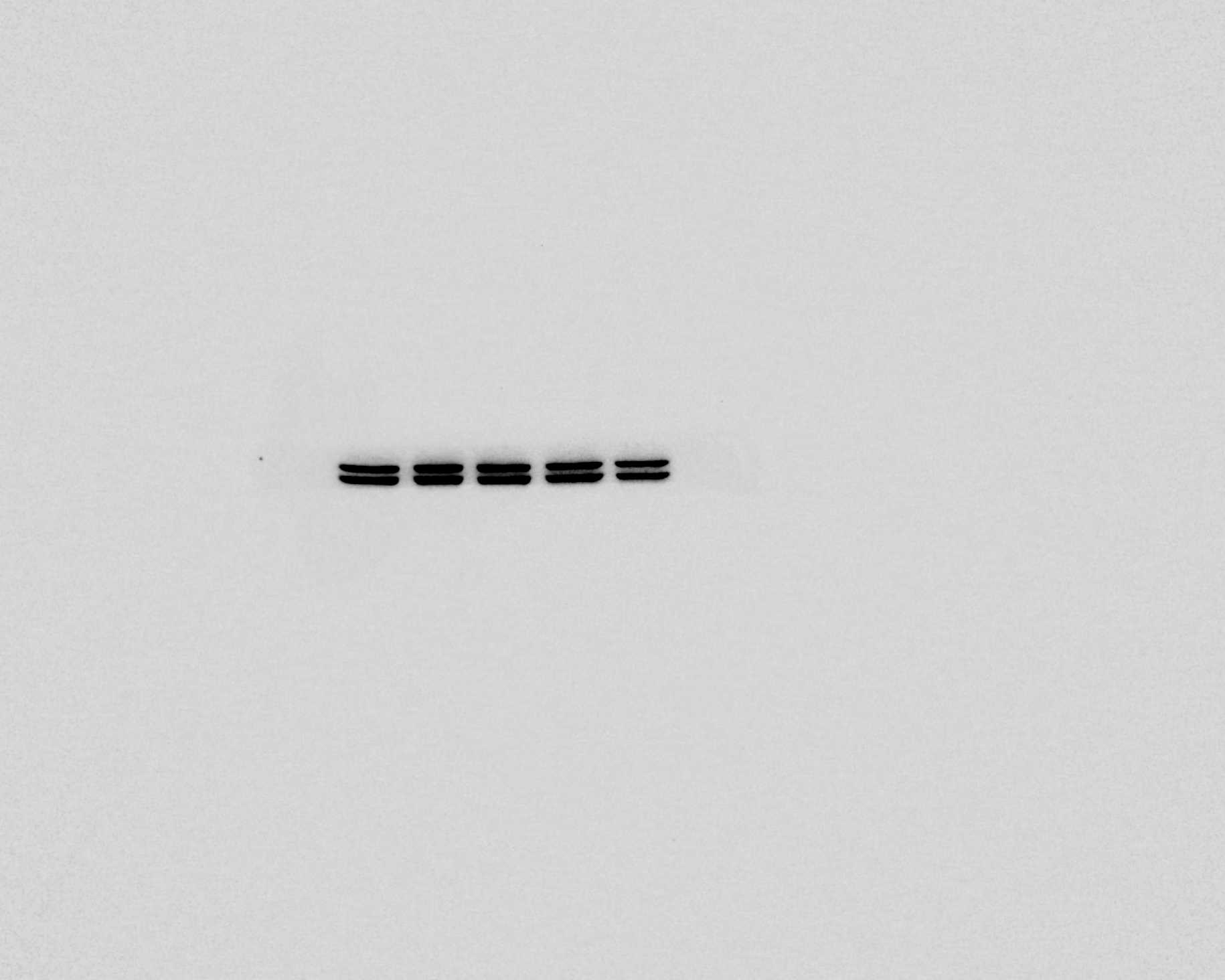

Supplement: Supplementary file 11 [file DataSheet2.ZIP › Weatern blot raw data 2/erk1/erk1.jpg]

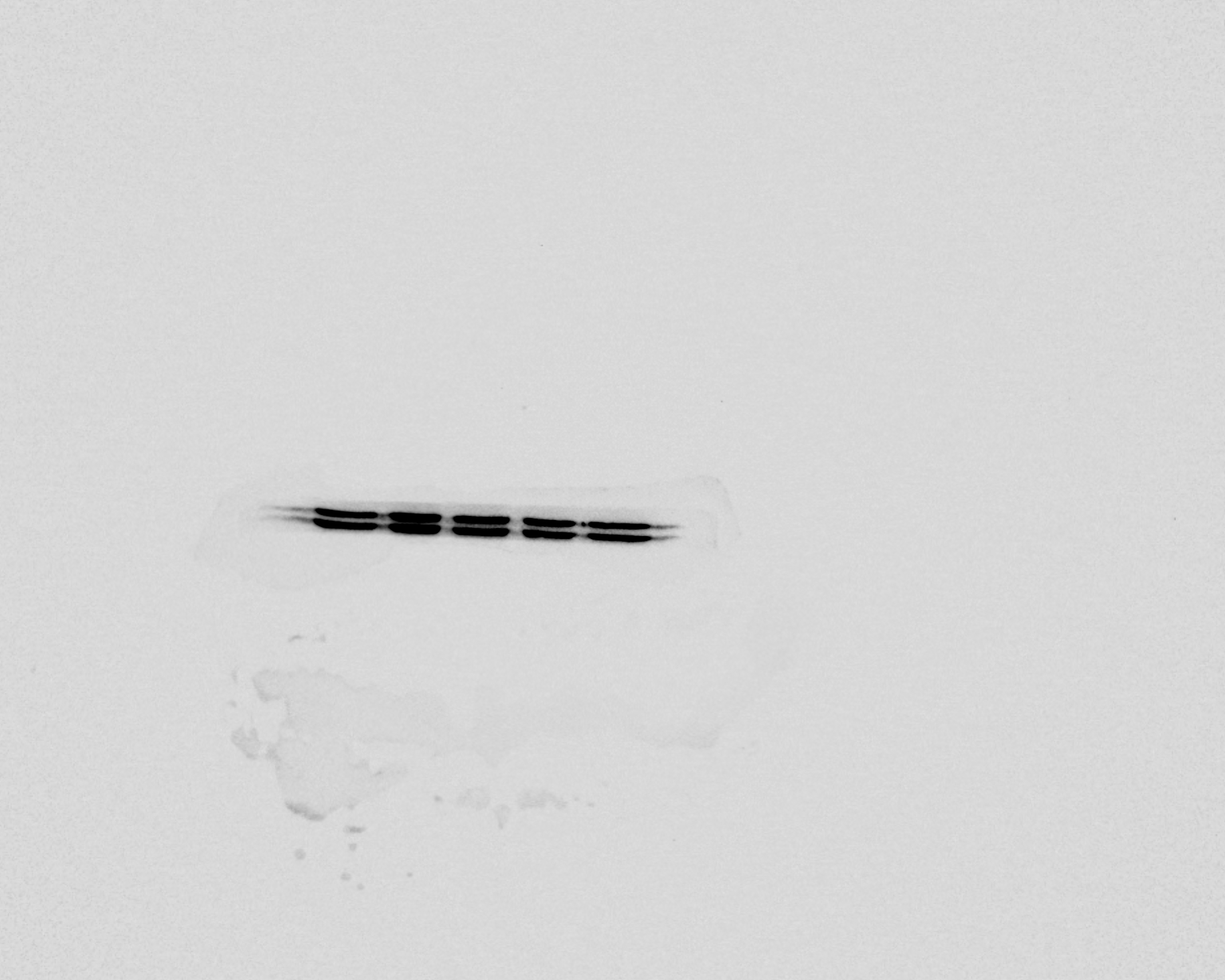

Supplement: Supplementary file 11 [file DataSheet2.ZIP › Weatern blot raw data 2/erk1/erk2.jpg]

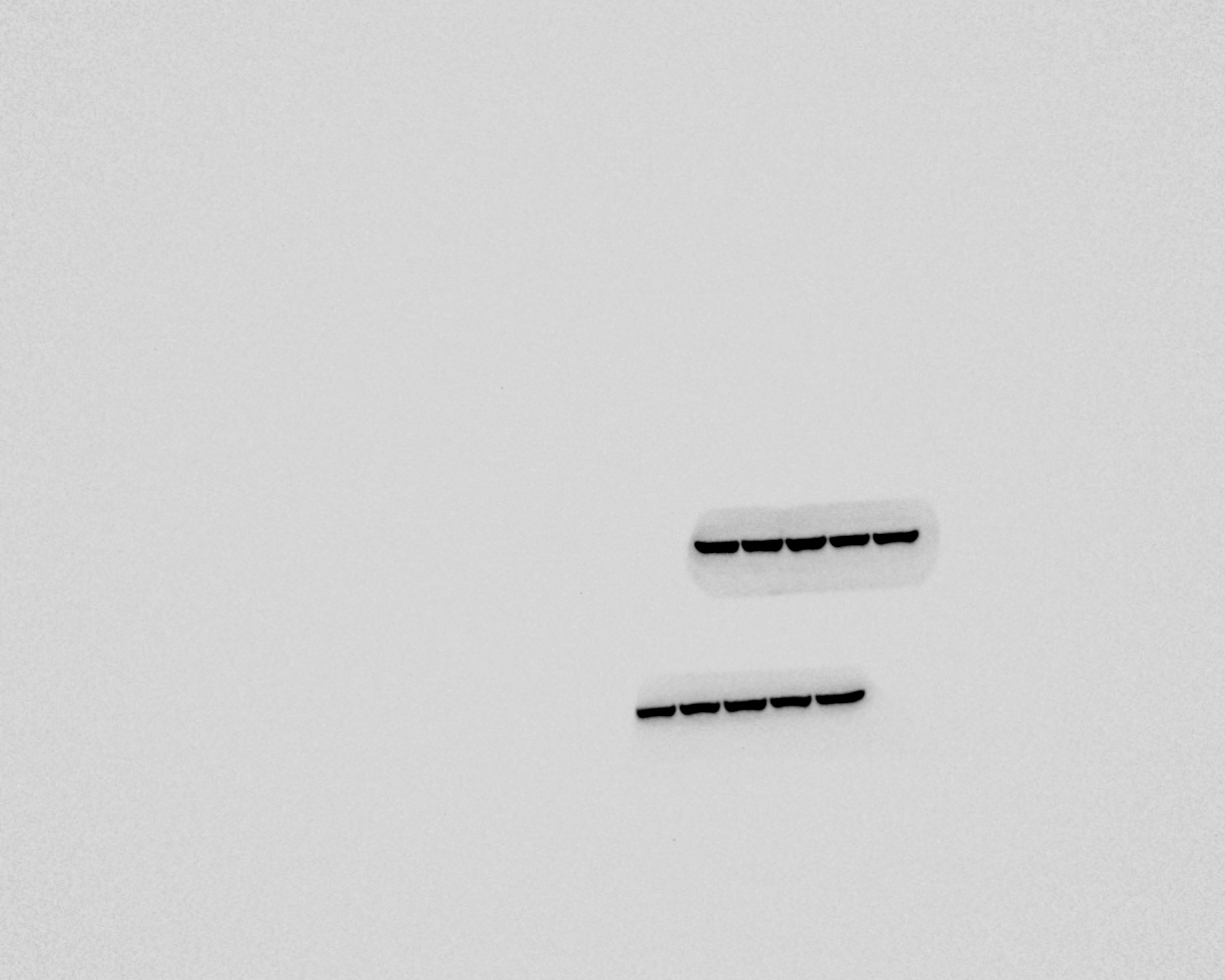

Supplement: Supplementary file 11 [file DataSheet2.ZIP › Weatern blot raw data 2/erk2/actin3+a.jpg]

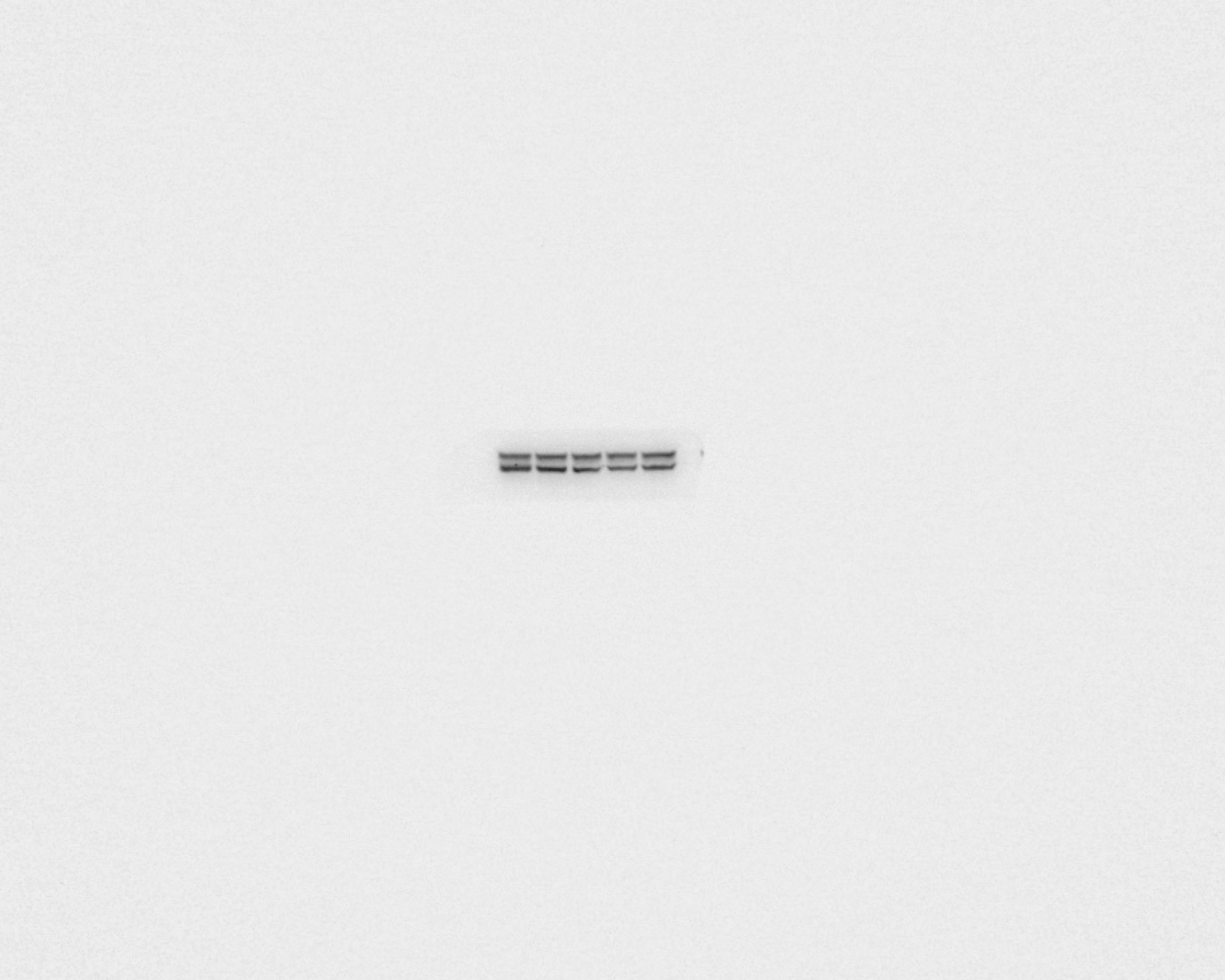

Supplement: Supplementary file 11 [file DataSheet2.ZIP › Weatern blot raw data 2/erk2/erk 3.jpg]

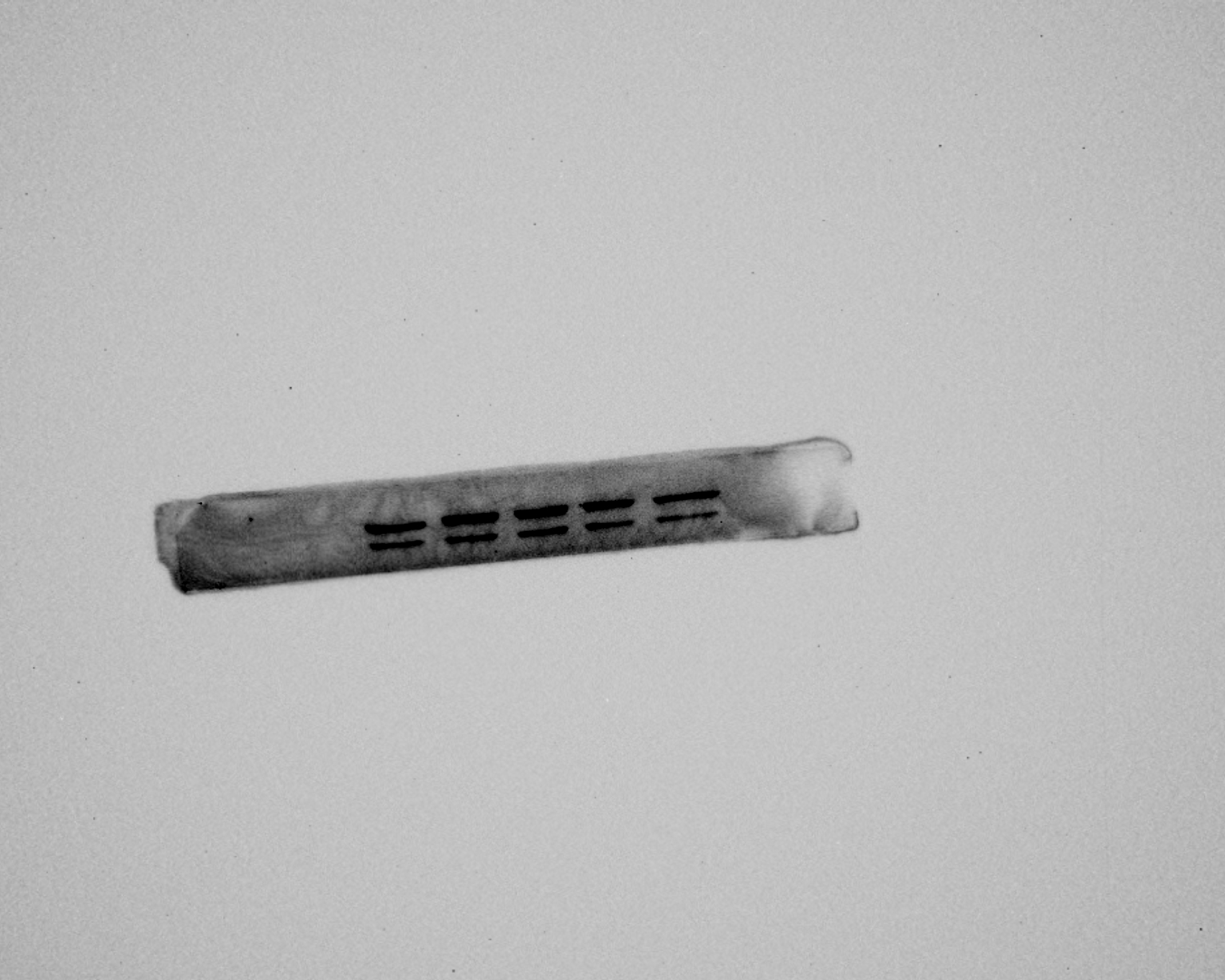

Supplement: Supplementary file 11 [file DataSheet2.ZIP › Weatern blot raw data 2/jnk/jnk 1.jpg]

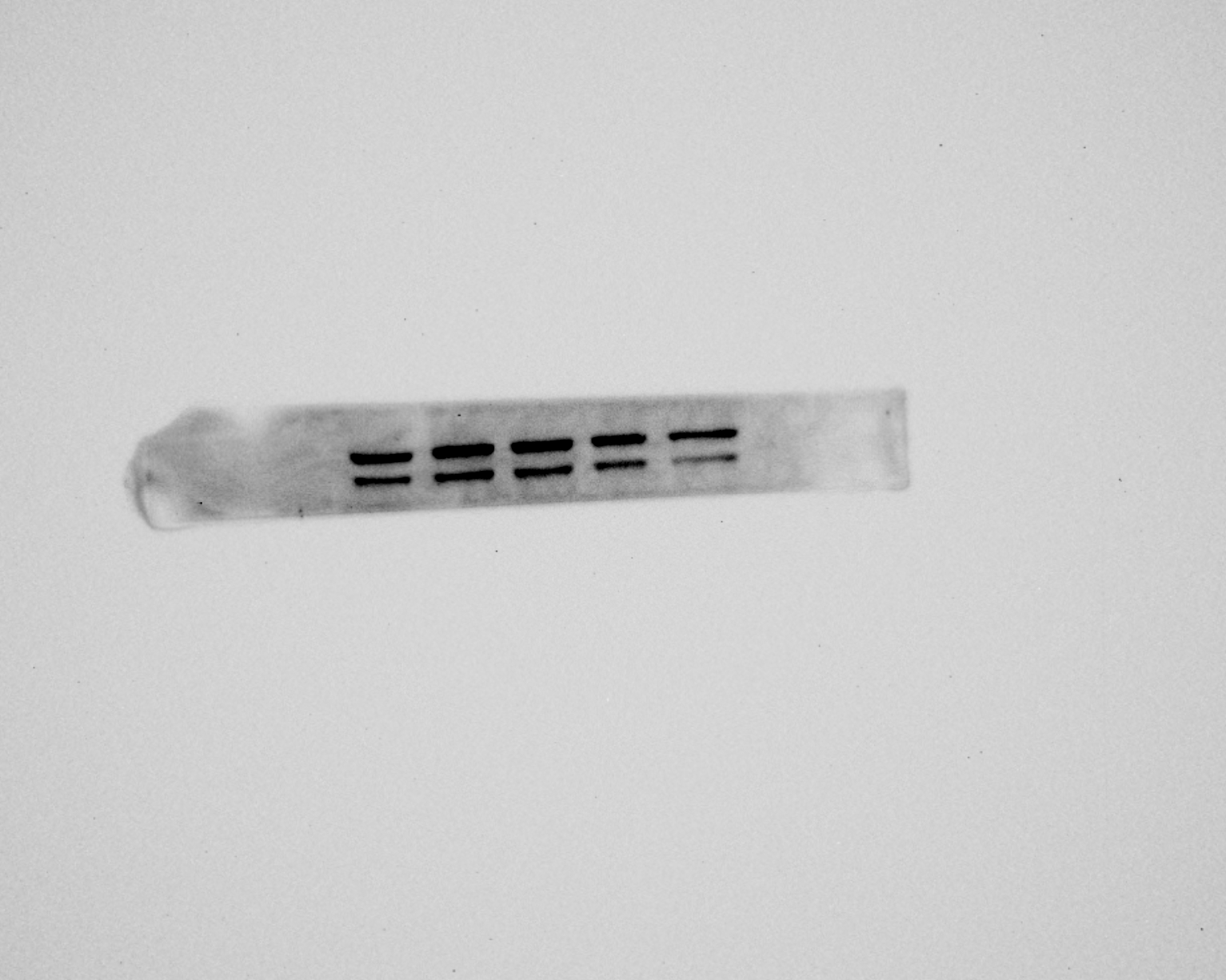

Supplement: Supplementary file 11 [file DataSheet2.ZIP › Weatern blot raw data 2/jnk/jnk 2.jpg]

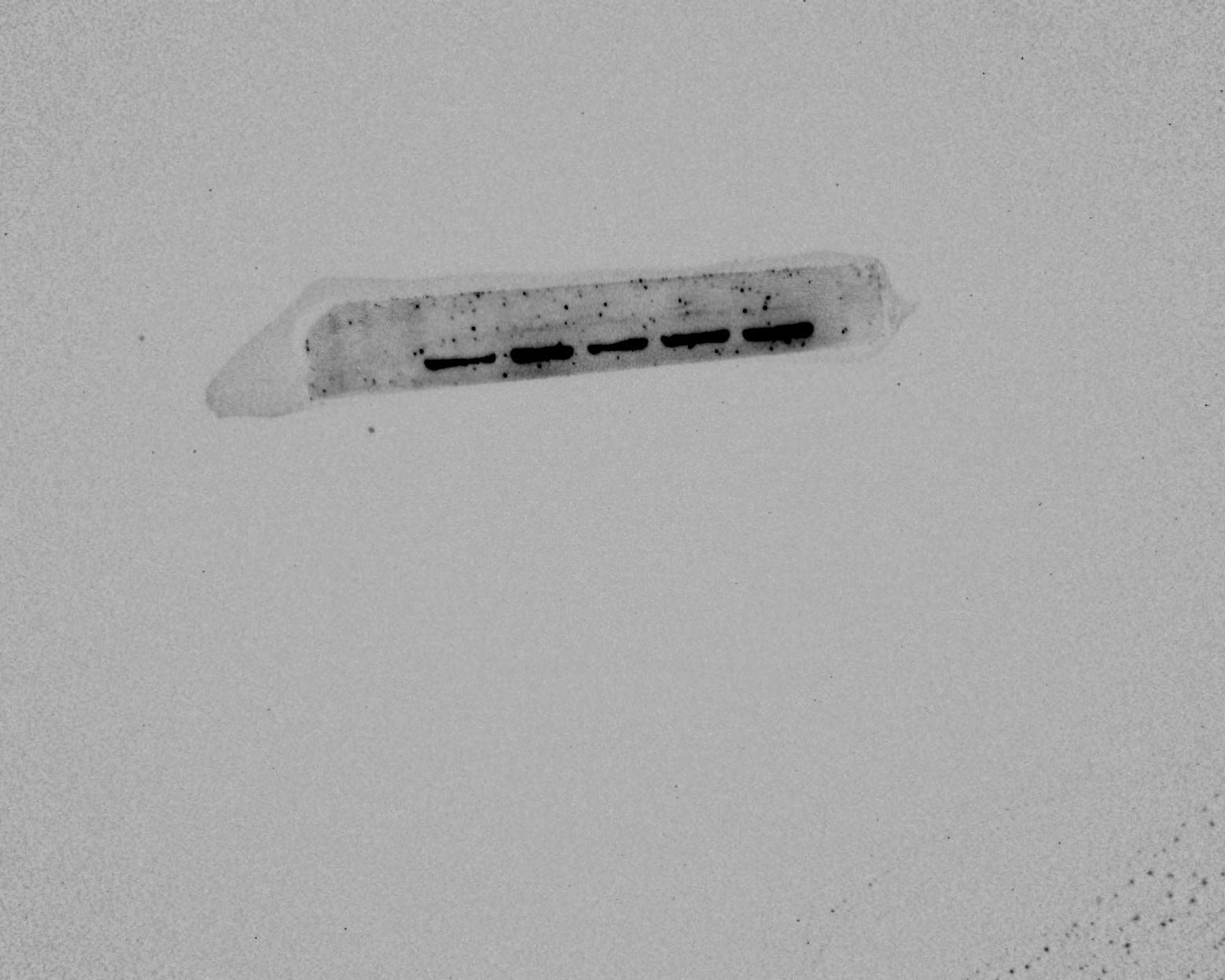

Supplement: Supplementary file 11 [file DataSheet2.ZIP › Weatern blot raw data 2/jnk/jnk3.jpg]

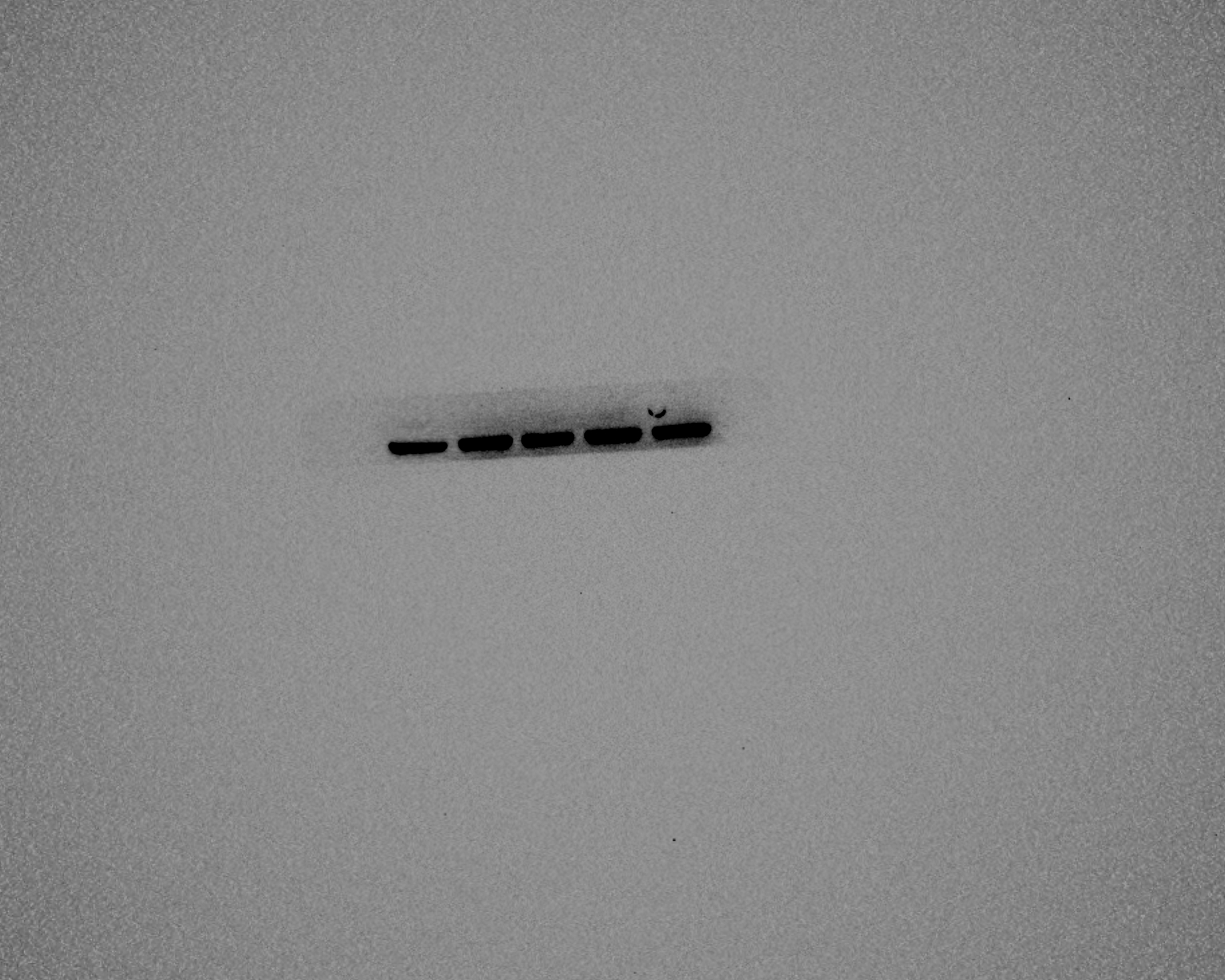

Supplement: Supplementary file 11 [file DataSheet2.ZIP › Weatern blot raw data 2/jnk/tubulin 3.jpg]

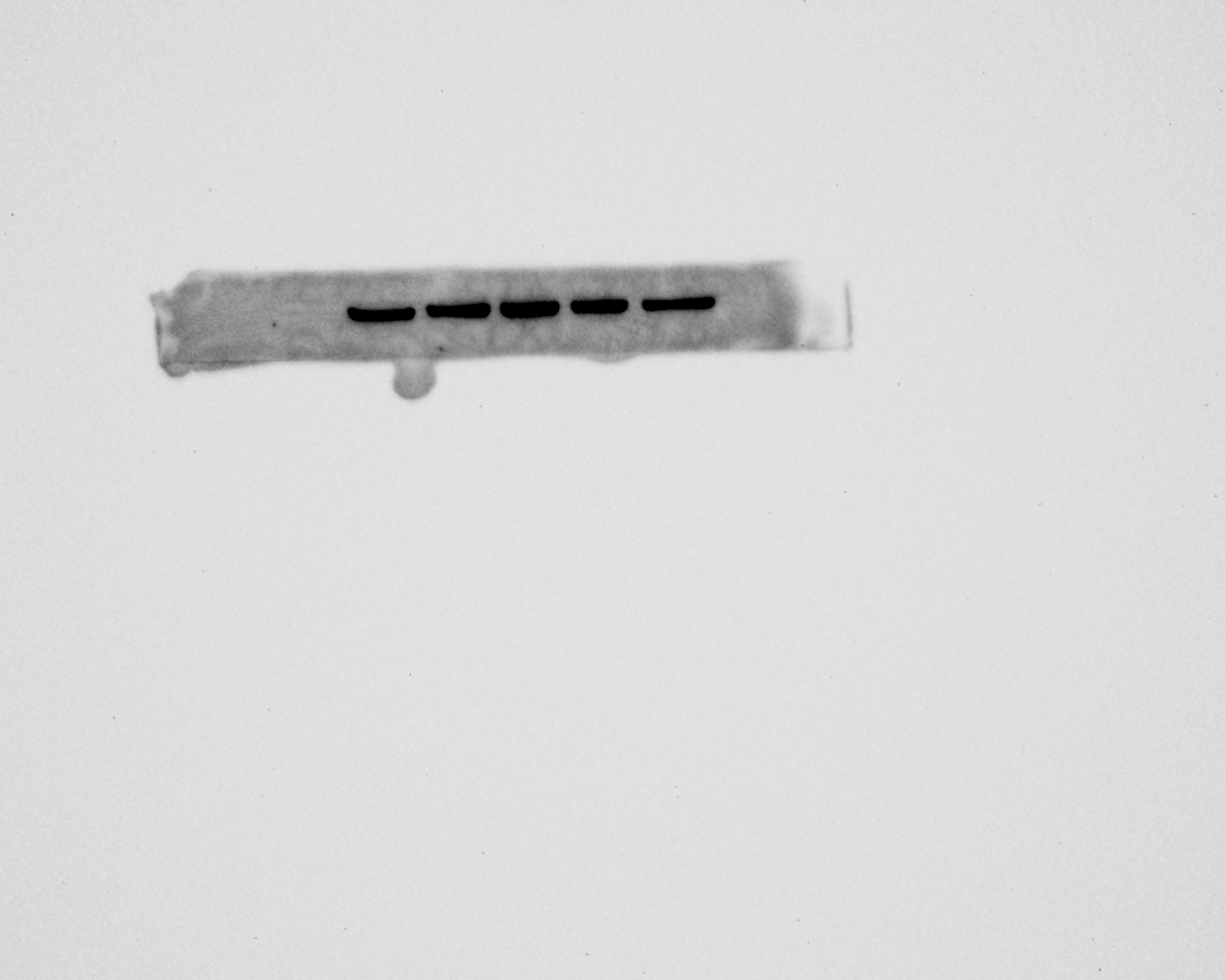

Supplement: Supplementary file 11 [file DataSheet2.ZIP › Weatern blot raw data 2/jnk/tubulin1.jpg]

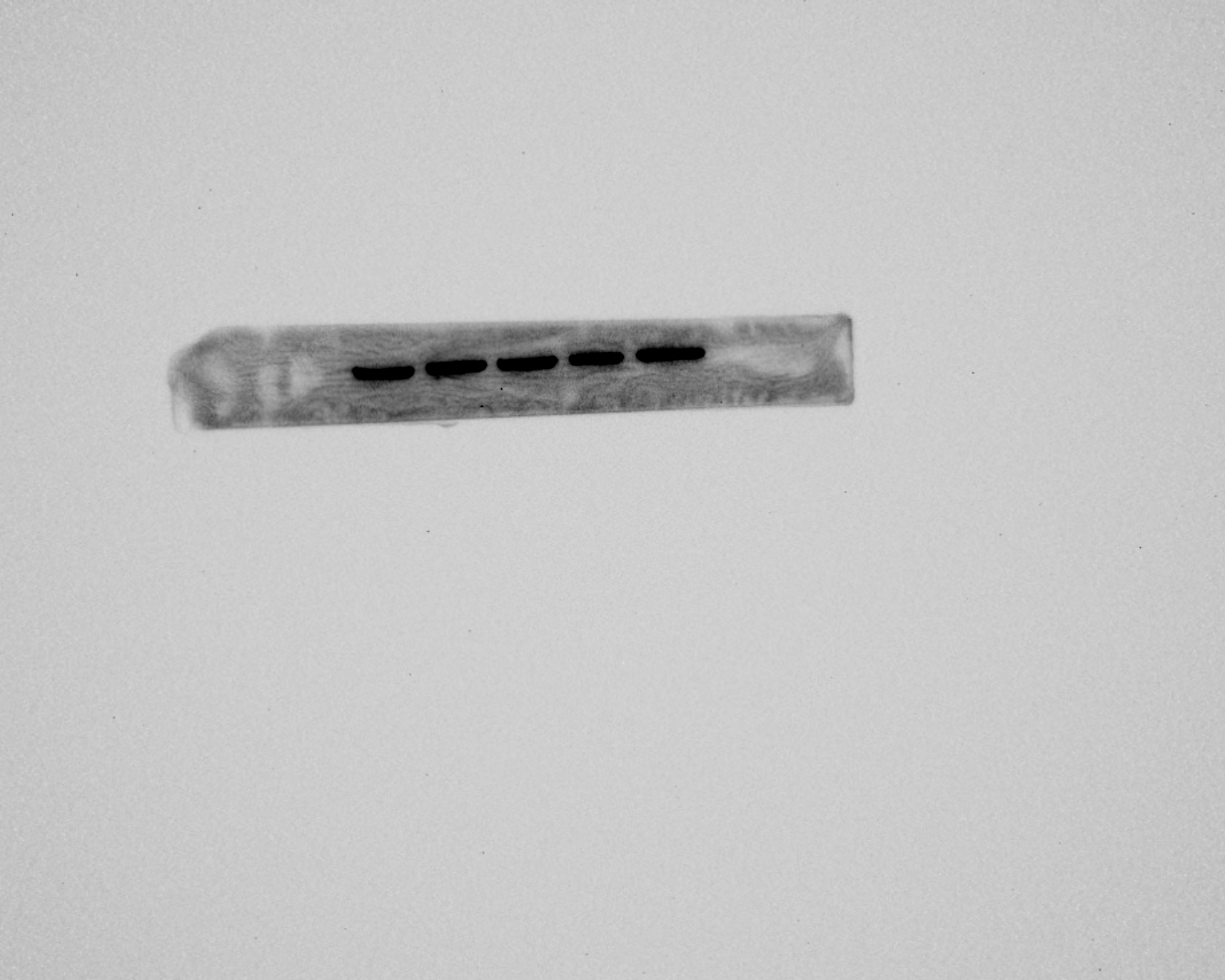

Supplement: Supplementary file 11 [file DataSheet2.ZIP › Weatern blot raw data 2/jnk/tubulin2.jpg]

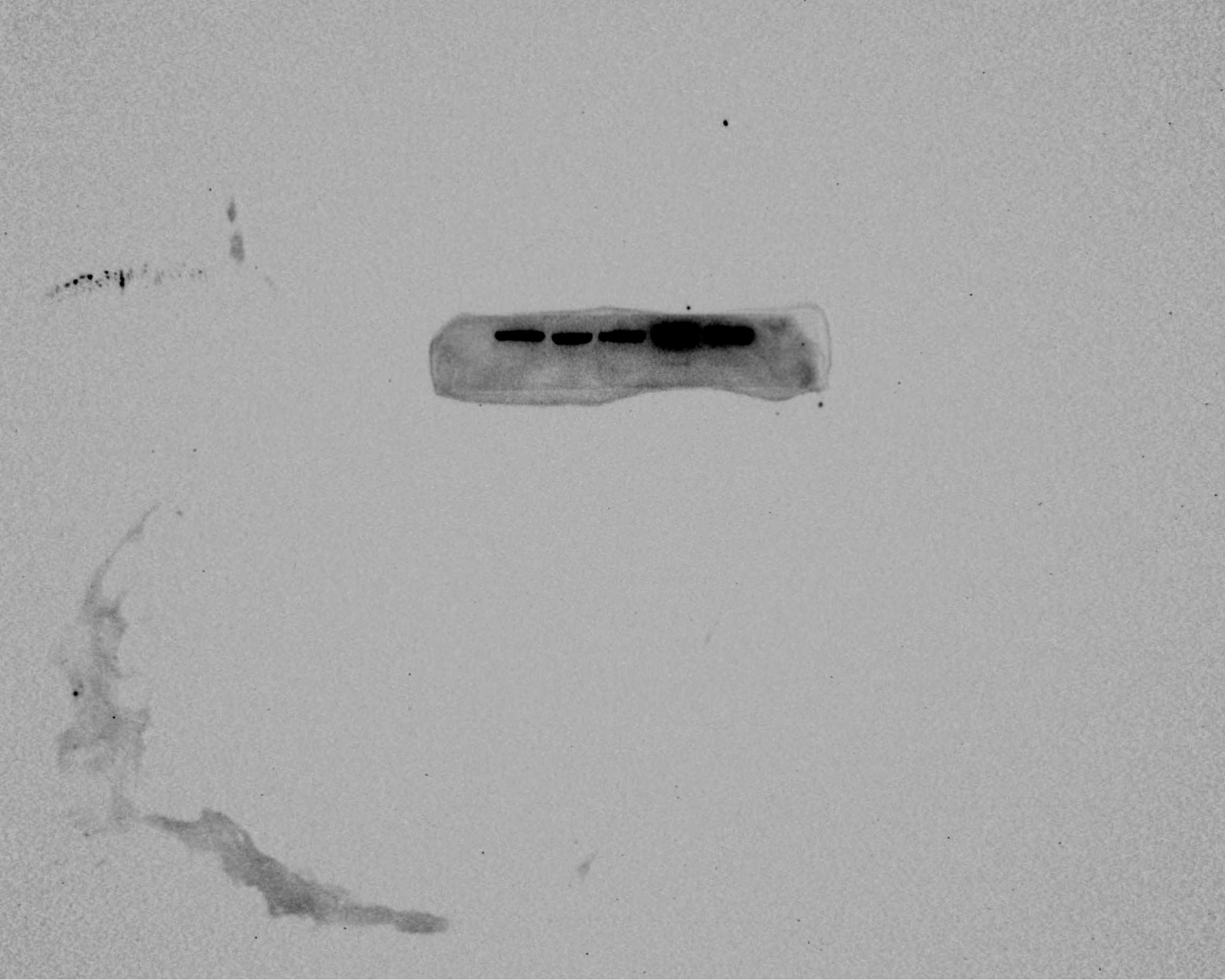

Supplement: Supplementary file 11 [file DataSheet2.ZIP › Weatern blot raw data 2/p38/p38 1.jpg]

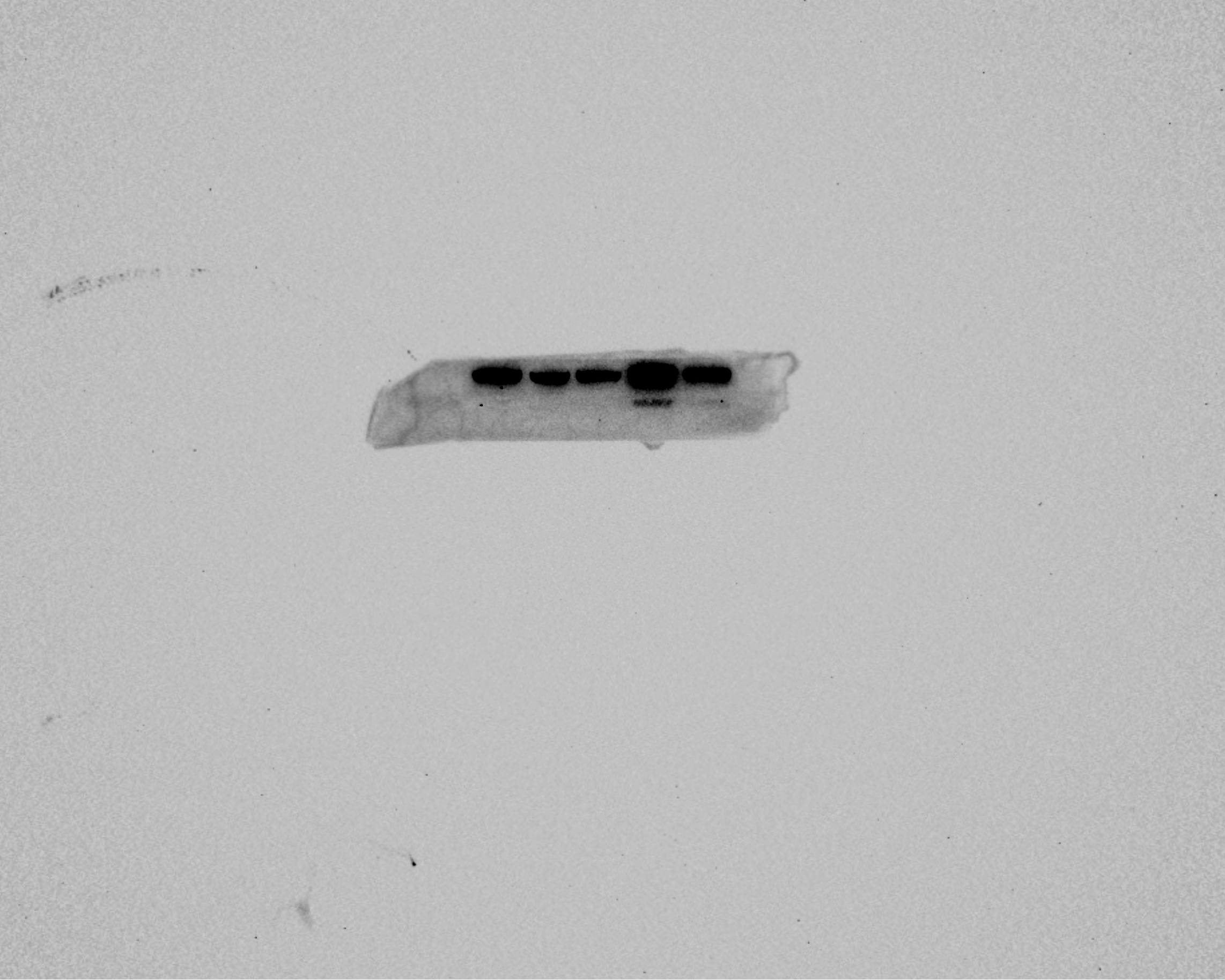

Supplement: Supplementary file 11 [file DataSheet2.ZIP › Weatern blot raw data 2/p38/p38 2.jpg]

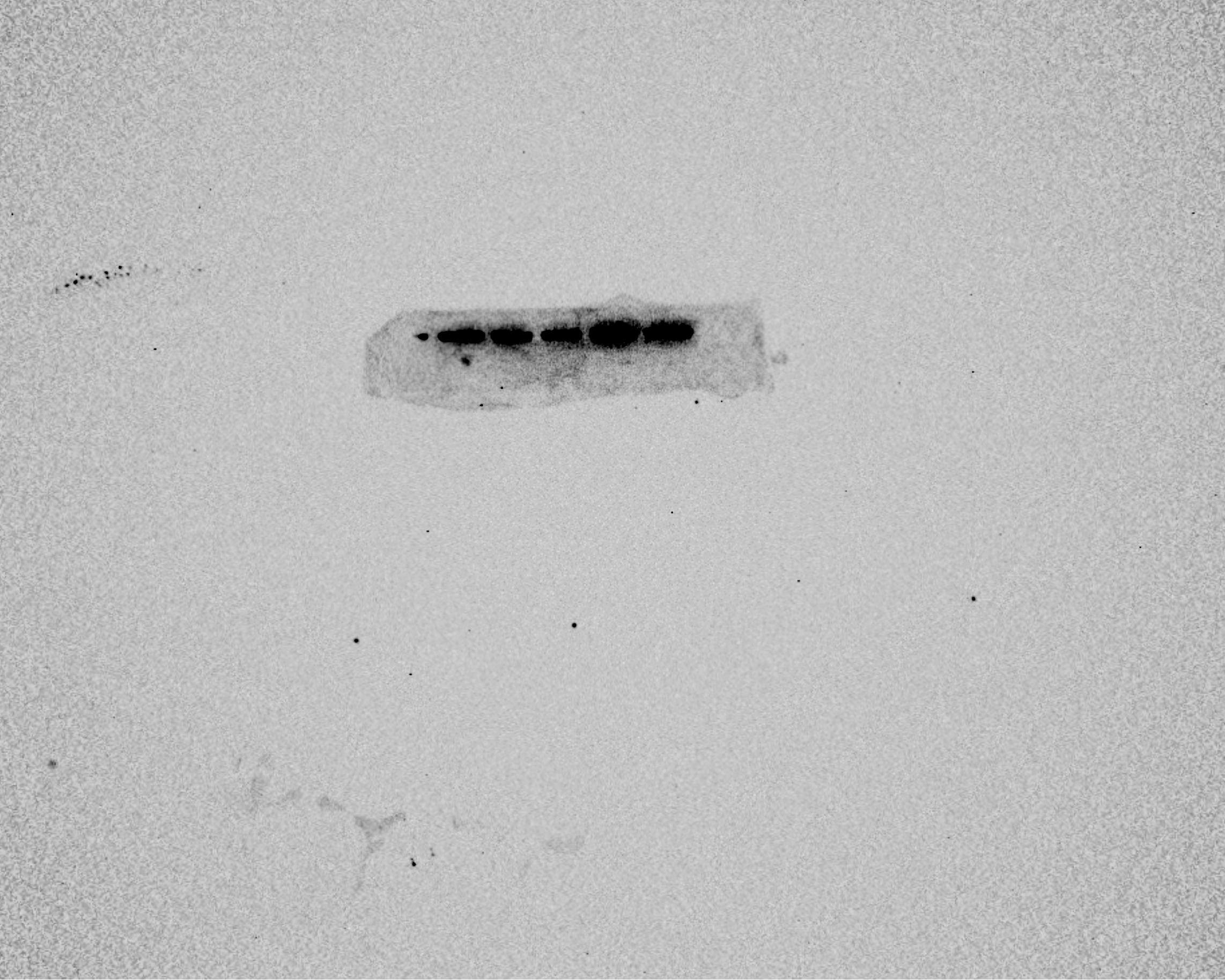

Supplement: Supplementary file 11 [file DataSheet2.ZIP › Weatern blot raw data 2/p38/p38 3.jpg]

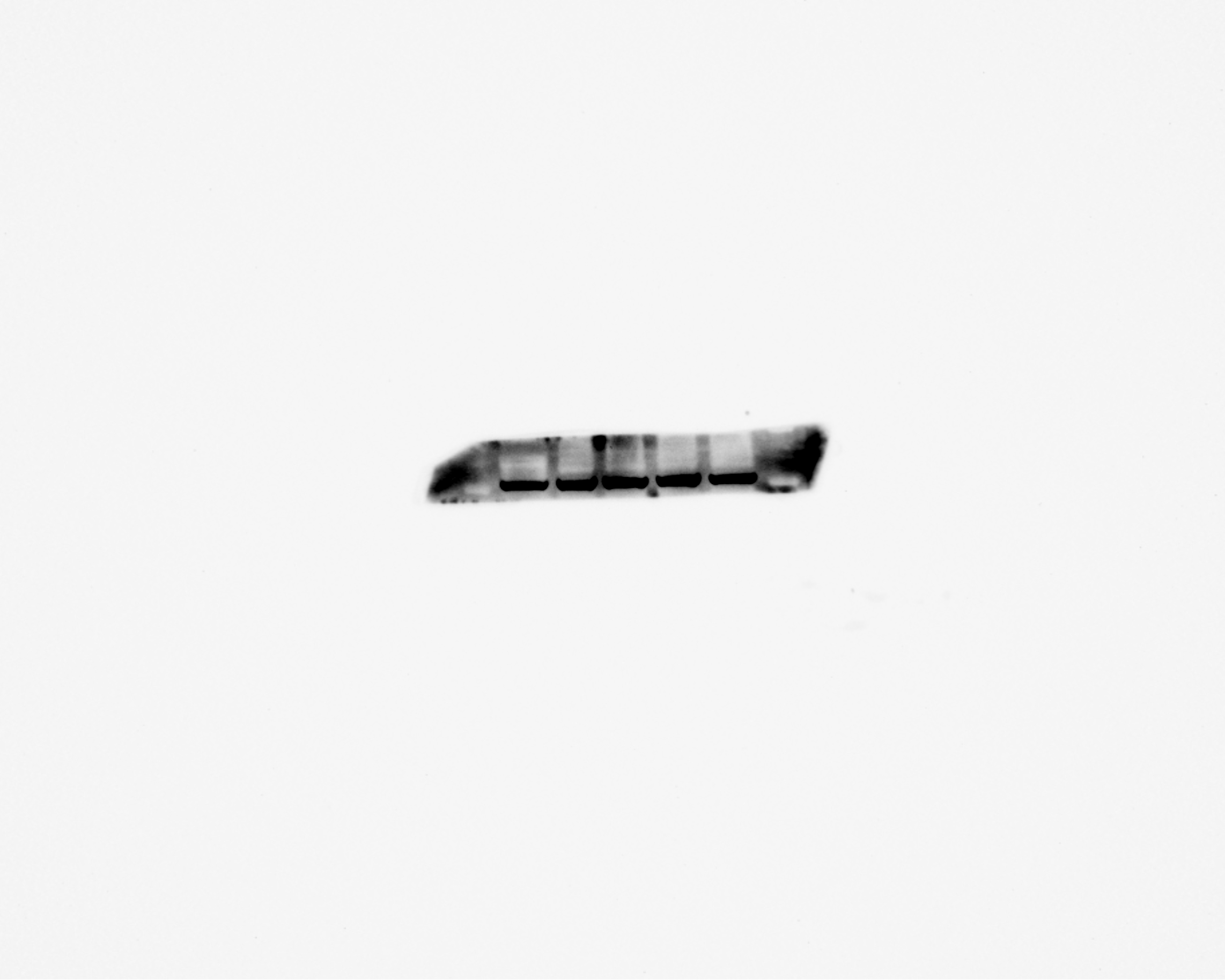

Supplement: Supplementary file 11 [file DataSheet2.ZIP › Weatern blot raw data 2/p38/tubulin 2.jpg]

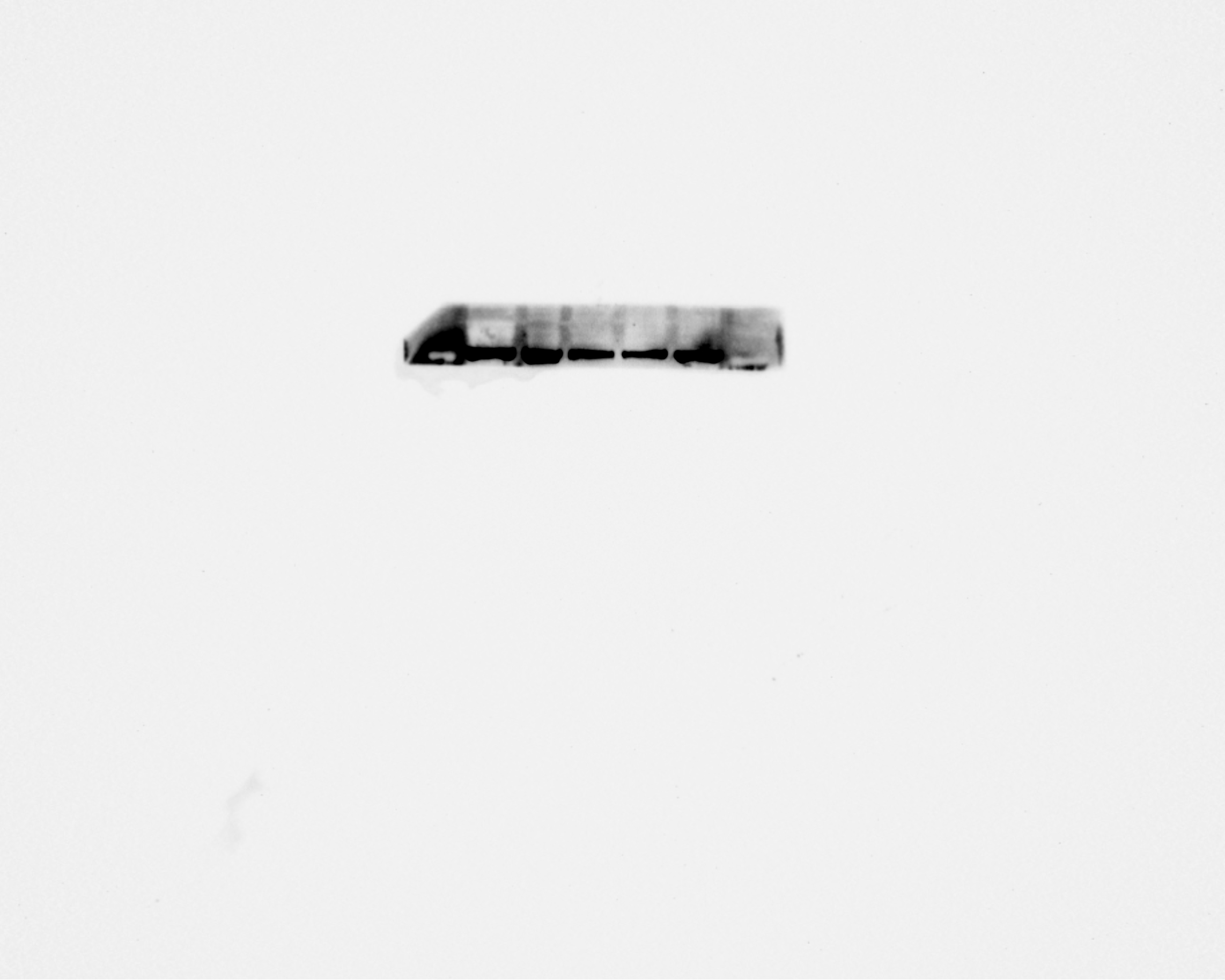

Supplement: Supplementary file 11 [file DataSheet2.ZIP › Weatern blot raw data 2/p38/tubulin1.jpg]

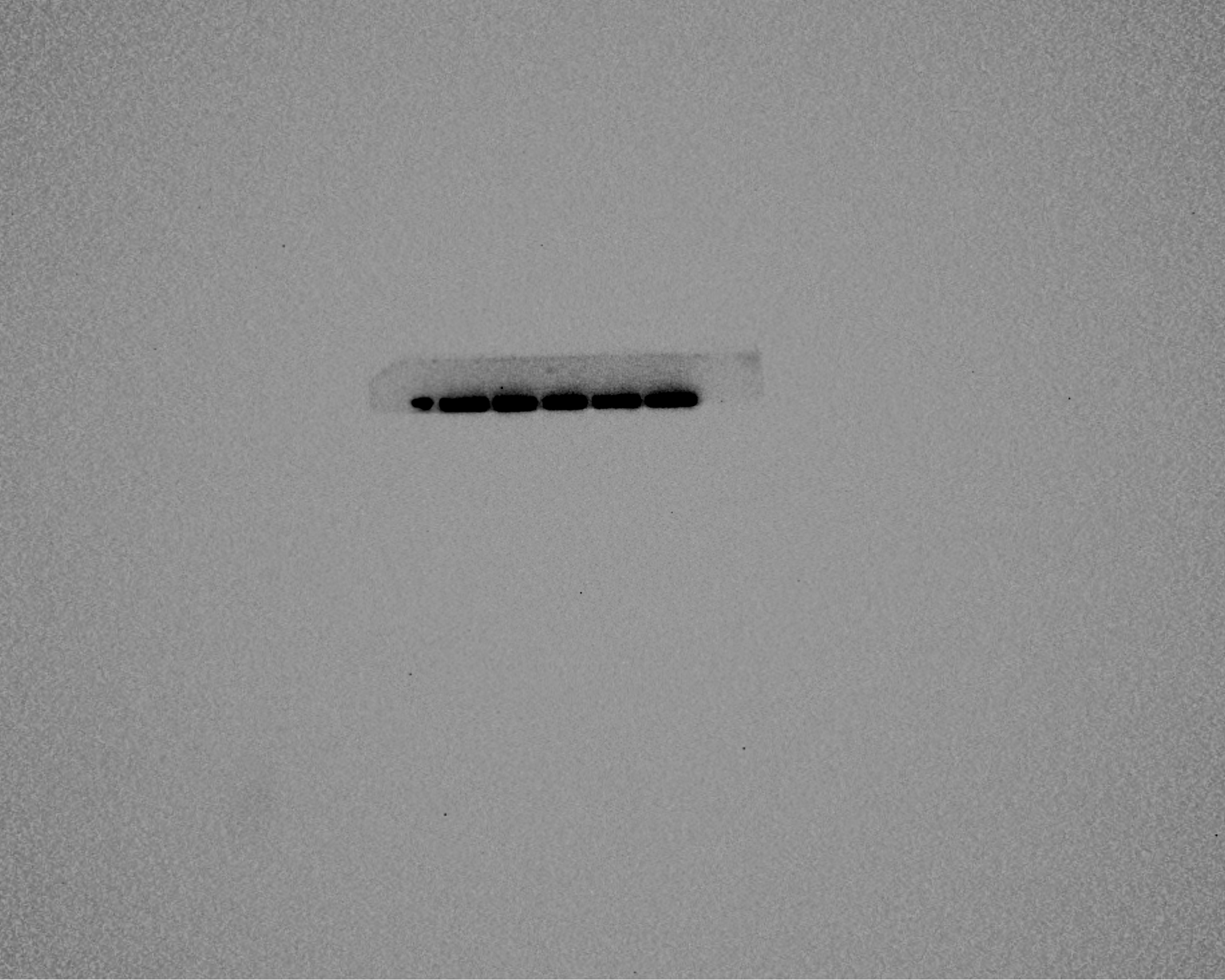

Supplement: Supplementary file 11 [file DataSheet2.ZIP › Weatern blot raw data 2/p38/tubulin3.jpg]
