# Supplementary material for: Mechanism and Protective Effect of Smilax glabra Roxb on the Treatment of Heart Failure via Network Pharmacology Analysis and Vitro Verification
Source: Front Pharmacol. 2022 May 23;13:868680. doi: 10.3389/fphar.2022.868680 (PMC9169610; doi:10.3389/fphar.2022.868680)
Supplement: Supplementary file 5 [file Presentation1.pptx]

## Slide 1
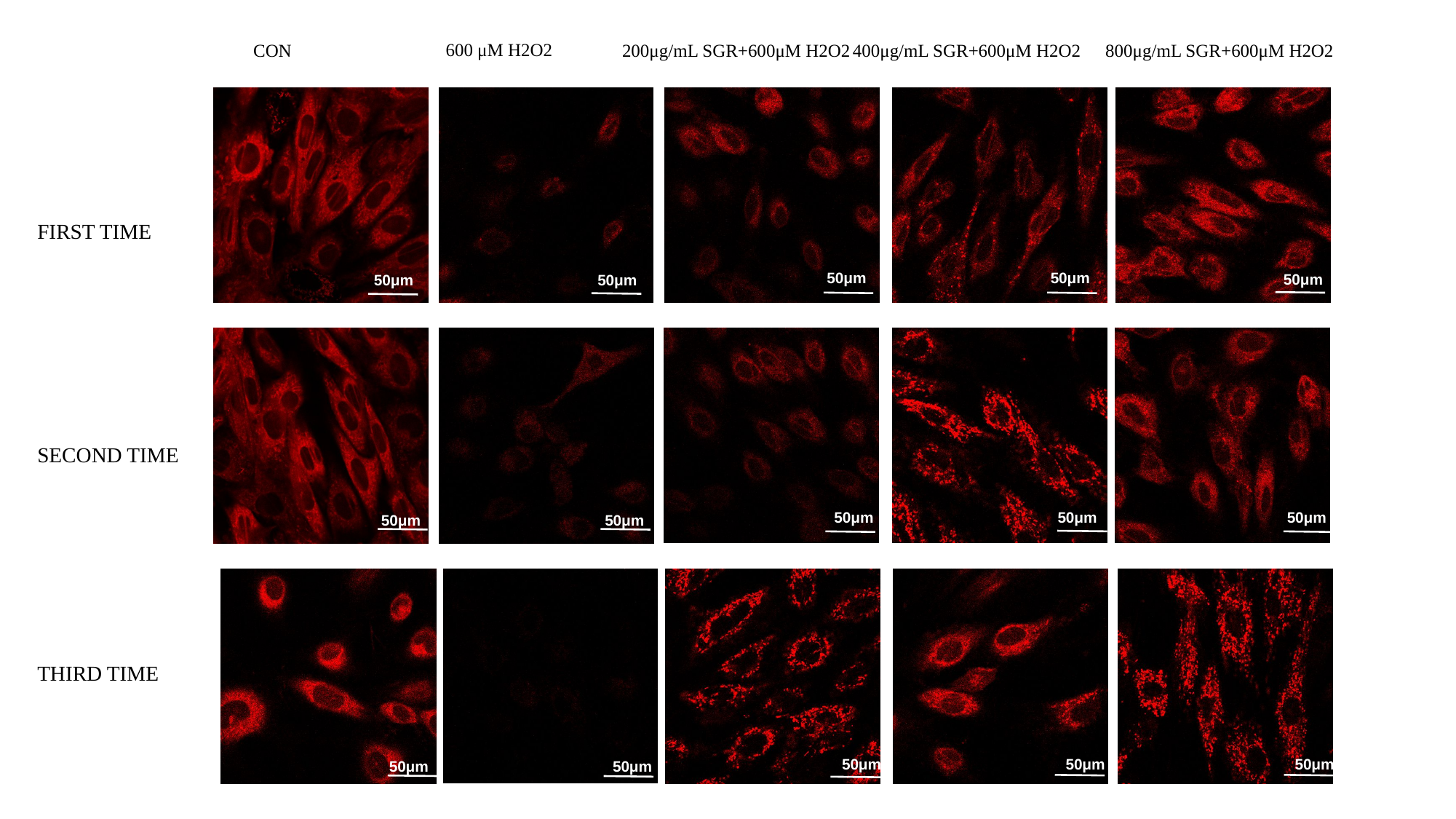

600 μM H2O2
CON
200μg/mL SGR+600μM H2O2
400μg/mL SGR+600μM H2O2
800μg/mL SGR+600μM H2O2
50μm
50μm
50μm
50μm
50μm
50μm
50μm
50μm
50μm
50μm
50μm
50μm
50μm
50μm
50μm
FIRST TIME
SECOND TIME
THIRD TIME
 Figure 7B: SGR protected mitochondrial damage induced by H2O2, presenting by mitochondrial membrane potential
