# Supplementary material for: Mechanism and Protective Effect of Smilax glabra Roxb on the Treatment of Heart Failure via Network Pharmacology Analysis and Vitro Verification
Source: Front Pharmacol. 2022 May 23;13:868680. doi: 10.3389/fphar.2022.868680 (PMC9169610; doi:10.3389/fphar.2022.868680)
Supplement: Supplementary file 12 [file Presentation3.PPTX]

## Slide 1
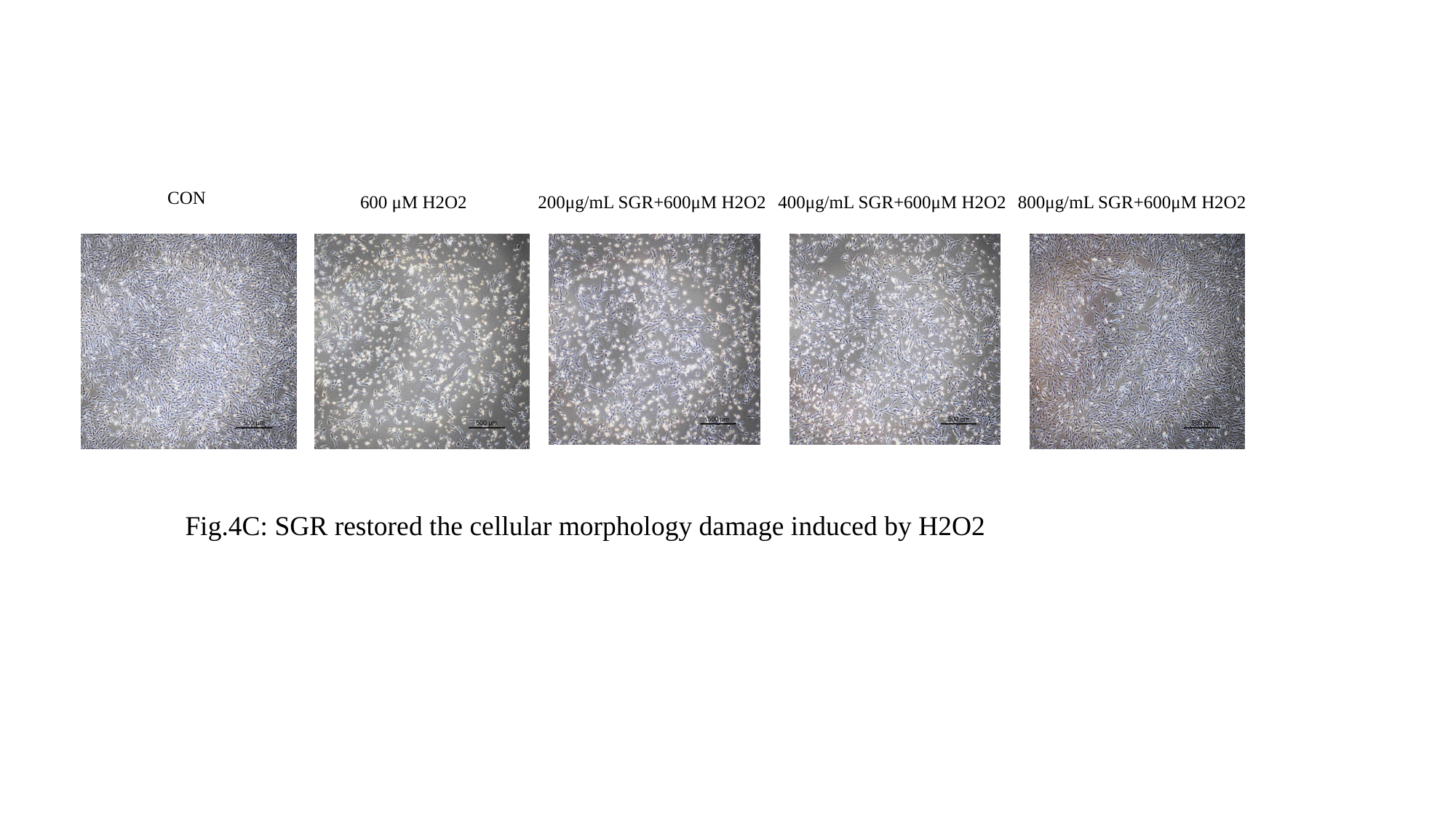

CON
600 μM H2O2
200μg/mL SGR+600μM H2O2
400μg/mL SGR+600μM H2O2
800μg/mL SGR+600μM H2O2
 Fig.4C: SGR restored the cellular morphology damage induced by H2O2

## Slide 2
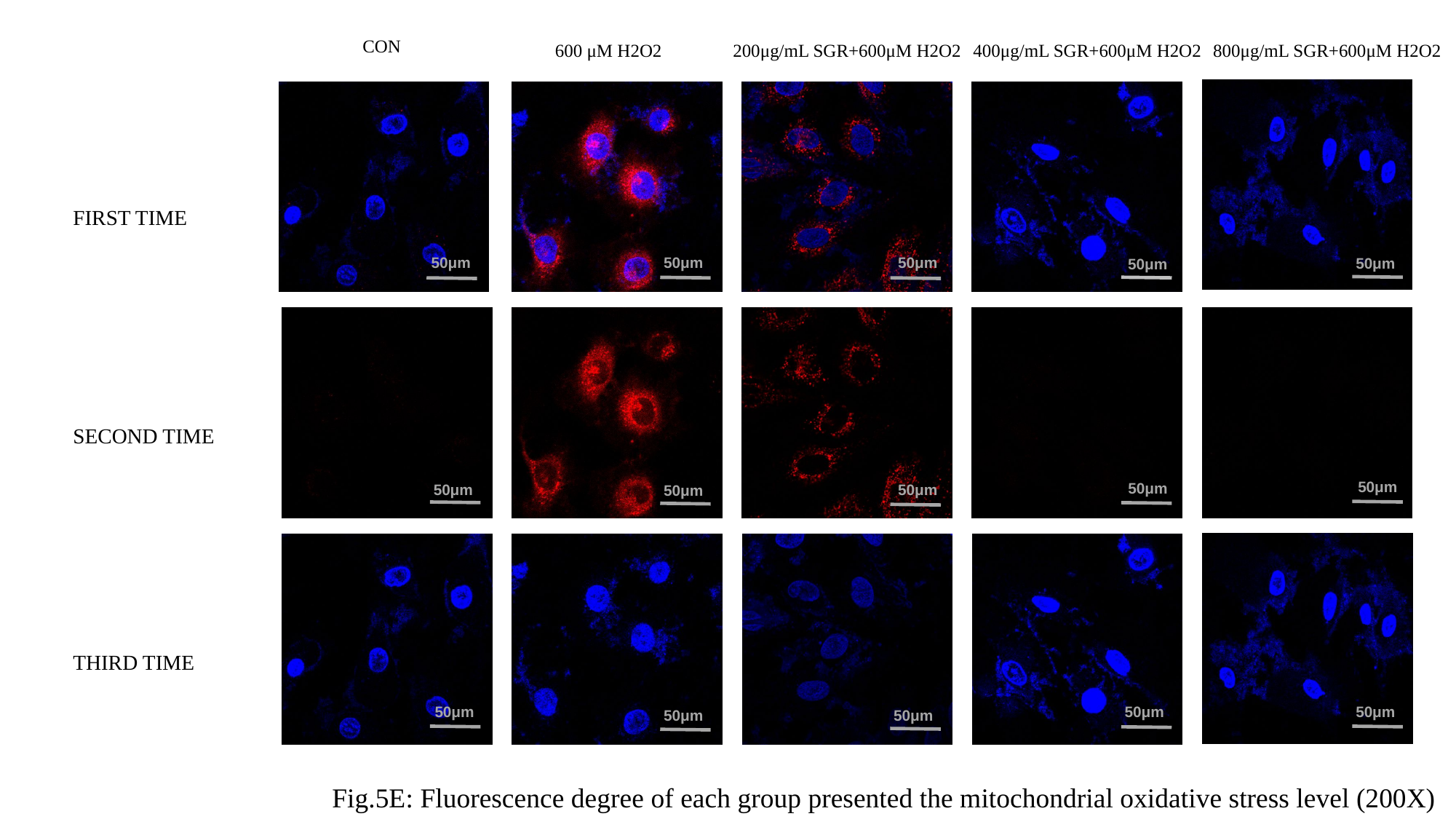

CON
600 μM H2O2
200μg/mL SGR+600μM H2O2
400μg/mL SGR+600μM H2O2
800μg/mL SGR+600μM H2O2
50μm
50μm
50μm
50μm
50μm
50μm
50μm
50μm
50μm
50μm
50μm
50μm
50μm
50μm
50μm
FIRST TIME
SECOND TIME
THIRD TIME
Fig.5E: Fluorescence degree of each group presented the mitochondrial oxidative stress level (200X)
